# Supplementary material for: Identification of Shared Neoantigens in BRCA1-Related Breast Cancer
Source: Vaccines (Basel). 2022 Sep 22;10(10):1597. doi: 10.3390/vaccines10101597 (PMC9609887; doi:10.3390/vaccines10101597)
Supplement: Supplementary file 1 [file vaccines-10-01597-s001.zip › vaccines-1858387-supplementary.pdf]

Supplementary Figure S1. Overlapping top 20 recurrent somatic mutations identified in *BRCA1*-positive and *BRCA1*-negative groups. The somatic mutations on the lists and in the tables are the overlapped recurrent somatic mutations from both subgroups. Its corresponding frequency (percentage of sample harboring the somatic mutation in all *BRCA1*-positive samples or *BRCA1*-negative samples) are also shown in the table for each database.

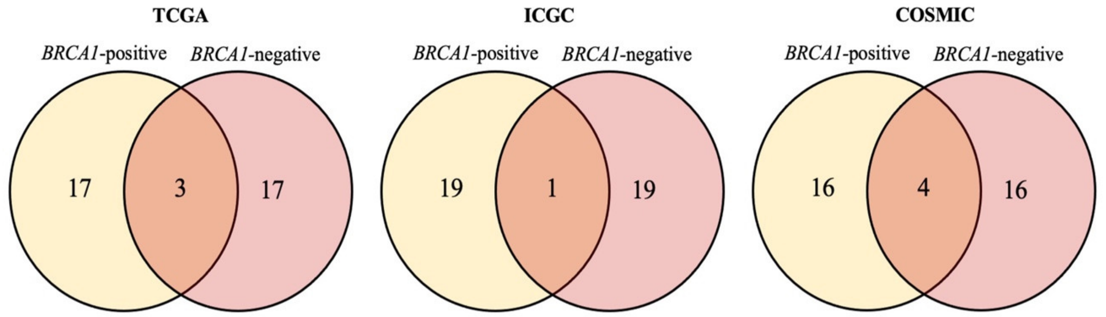

Overlapping somatic mutations :

TCGA: *PIK3CA* H1047R, *TP53* R196*, *GATA3* D336Gfs*17

ICGC: *PIK3CA* E542K

COSMIC: *PIK3CA* H1047R, *PIK3CA* E542K, *PIK3CA* E545K, *GATA3* D336Gfs*17

TCGA	*BRCA1*-positive	*BRCA1-negative*
PIK3CA H1047R	16.67%	12.19%
TP53 R196*	8.33%	1.62%
GATA3 D336Gfs*17	8.33%	2.43%

ICGC	*BRCA1*-positive	*BRCA1-negative*
PIK3CA E542K	6.67%	3.96%

COSMIC	*BRCA1*-positive	*BRCA1-negative*
PIK3CA H1047R	4.54%	15.45%
PIK3CA E542K	4.54%	4.78%
PIK3CA E545K	1.51%	7.63%
GATA3 D336Gfs*17	3.03%	0.56%

Supplementary Table S1. Sample characteristics of germline *BRCA1*-mutated breast cancer studies and sample IDs of the samples included in this study.

	**Nik-Zanial et al. 2016**	**Nones et al. 2019**	**Inagaki-Kawata et al. 2020**
**Samples**	31	26	21
**Analysis type**	WGS	WGS	Target sequencing of 115 genes associated with breast cancer
**Platform**	Illumina Hiseq 2000/2500	Illumina X-Ten	SureSelect system (Agilent)
**Raw data available at**	EGAS00001001178 (the European-Genome Phenome Archive)	EGAS00001003305 (the European-Genome Phenome Archive)	EGAS00001004630, EGAS00001004182 (the European-Genome Phenome Archive)
**Sample IDs**	PD24202a<br>PD8621a<br>PD13296a<br>PD24337a<br>PD9702a<br>PD6406a<br>PD9004a<br>PD3905a<br>PD24186a<br>PD4005a<br>PD13771a<br>PD13297a<br>PD5935a<br>PD11327a<br>PD10014a<br>PD3890a<br>PD5930a<br>PD8980a<br>PD14442a<br>PD4006a<br>PD13299a<br>PD4107a<br>PD23562a<br>PD5945a<br>PD22355a<br>PD23574a<br>PD23578a<br>PD6413a<br>PD6731a2<br>PD5948a<br>PD13627a	FBC108061<br>FBC110004<br>FBC110062<br>FBC112021<br>FBC208006<br>FBC712007<br>FBC406015<br>FBC016026<br>FBC020030<br>FBC061126<br>FBC020187<br>FBC070197<br>FBC100739<br>FBC020675<br>FBC070890<br>FBC060097<br>FBC060467<br>FBC020034<br>FBC070205<br>FBC060031<br>FBC020091<br>FBC030130<br>FBC061699<br>FBC071015<br>FBC080607<br>FBC090326	TCGA-D8-A1XK<br>TCGA-D8-A1XQ<br>TCGA-AR-A24Q<br>TCGA-AO-A124<br>TCGA-A7-A4SE<br>TCGA-D8-A147<br>TCGA-C8-A12K<br>TCGA-E2-A14N<br>TCGA-E9-A244<br>TCGA-E9-A22G<br>TCGA-A1-A0SH<br>TCGA-E9-A22E<br>TCGA-E2-A14Z<br>TCGA-EW-A1P4<br>TCGA-E2-A1IJ<br>TCGA-BH-A0BL<br>KU045<br>KU016<br>KU014<br>KU009<br>KU085

Supplementary Table S2. *BRCA1*-positive and -negative sample IDs from TCGA, ICGC and COSMIC databases.

TCGA: *BRCA1*- positive

Sample ID	DNA Change(BRCA1 mutation)	BRCA1 mutation type	ACMG prediction	GRCh
TCGA-BH-A0WA	chr17:g.43071239C>T	Splice Acceptor BRCA1 X1580_splice	pathogenic	38
TCGA-A1-A0SI	chr17:g.43124016C>T	Splice Donor BRCA1 X27_splice	pathogenic	38
TCGA-A2-A25B	chr17:g.43093373C>A	Stop Gained BRCA1 E720*	pathogenic	38
TCGA-A1-A0SH	chr17:g.43092731G>A	Stop Gained BRCA1 Q934*	pathogenic	38
TCGA-A7-A6VW	chr17:g.43092822delA	Frameshift BRCA1 C903Wfs*97	pathogenic	38
TCGA-A7-A26H	chr17:g.43093467delTGTC	Frameshift BRCA1 T688Vfs*12	pathogenic	38
TCGA-LL-A8F5	chr17:g.43082539G>A	Stop Gained BRCA1 Q1408*	pathogenic	38
TCGA-A8-A06X	chr17:g.43094717C>A	Stop Gained BRCA1 E272*	pathogenic	38
TCGA-B6-A0X1	chr17:g.43124030delC	Frameshift BRCA1 E23Sfs*8	pathogenic	38
05BR052	chr17:g.43092073delA	Frameshift BRCA1 L1153Rfs*2	pathogenic	38
TCGA-E9-A1NC	chr17:g.43104148G>A	Stop Gained BRCA1 Q139*	pathogenic	38
TCGA-D8-A27M	chr17:g.43071073delG	Frameshift BRCA1 P1635Qfs*19	likely pathogenic	38
TCGA-AN-A0XU	chr17:g.43049164C>A	Missense BRCA1 G1809V	pathogenic	38

ICGC: *BRCA1*- positive

Sample ID	DNA change (BRCA1 mutation)	ACMG prediction	GRCh
DO225079	chr17:g.41234451G>A	Pathogenic	37
DO224917	chr17:g.41246005C>A	Pathogenic	37
DO225334	chr17:g.41247865->T	Likely Pathogenic	37
DO225328	chr17:g.41215381T>C	Likely Pathogenic	37
DO218669	chr17:g.41209130T>A	Pathogenic	37
DO1261	chr17:g.41244748G>A	Pathogenic	37
DO1712	chr17:g.41201181C>A	Pathogenic	37
DO2020	chr17:g.41276033C>T	Pathogenic	37
DO3916	chr17:g.41244185->T	Likely Pathogenic	37
DO50068	chr17:g.41234556G>A	Pathogenic	37
DO51226	chr17:g.41243563C>A	Pathogenic	37
DO5074	chr17:g.41246734C>A	Pathogenic	37
DO5130	chr17:g.41199695T>A	Likely Pathogenic	37
DO2897	chr17:g.41223256C>T	Pathogenic	37
DO2706	chr17:g.41245390C>A	Pathogenic	37

Comsic: *BRCA1*- positive

Sample ID	AA change (BRCA1 mutation)	CDS Mutation	ACMG prediction	GRCh
1509149	p.E515*	c.1543G>T	pathogenic	38
1527353	p.D1760V	c.5279A>T	pathogenic	38
1649378	p.R1443*	c.4327C>T	pathogenic	38
1660108	p.?	c.135-1215C>G	pathogenic	38
1768176	p.F1793Lfs*29	c.5356_5378dup	pathogenic	38
1779256	p.Q934*	c.2800C>T	pathogenic	38
1779260	p.?	c.80+1G>A	pathogenic	38
1779502	p.N1121Kfs*12	c.3362dup	likely pathogenic	38
1779649	p.?	c.4739-1G>A	pathogenic	38
1899647	p.E720*	c.2158G>T	pathogenic	38
1899672	p.E272*	c.814G>T	pathogenic	38
1899768	p.G1809V	c.5426G>T	pathogenic	38
1899917	p.Q1832L	c.5495A>T	likely pathogenic	38
1900097	p.P1635Qfs*19	c.4904del	likely pathogenic	38
2199791	p.Q1742R	c.5225A>G	likely pathogenic	38
2213140	p.A224Gfs*4	c.668dup	likely pathogenic	38
2262906	p.Q1408*	c.4222C>T	pathogenic	38
2318501	p.S1817Lfs*34	c.5448_5449insC	likely pathogenic	38
2339544	p.E1329*	c.3985G>T	pathogenic	38
2579117	p.D1526Mfs*43	c.4575del	likely pathogenic	38
2657324	p.E1419*	c.4255G>T	pathogenic	38
2662750	p.R1747Kfs*5	c.5240_5241del	pathogenic	38
2673932	p.C61Y	c.182G>A	pathogenic	38
2697837	p.R1610C	c.4828C>T	pathogenic	38
2697863	p.W1836*	c.5507G>A	pathogenic	38
2724995	p.R71Kfs*10	c.211dup	pathogenic	38
2725854	p.G911Efs*89	c.2732del	likely pathogenic	38
2726190	p.K1745*	c.5233A>T	pathogenic	38
2767653	p.E1250*	c.3748G>T	pathogenic	38
2767723	p.E1752Q	c.5254G>C	likely pathogenic	38
2768049	p.G928Afs*72	c.2783del	pathogenic	38
2768180	p.R1856Q	c.5567G>A	likely pathogenic	38
2768268	p.K608Ifs*3	c.1823_1826del	pathogenic	38
2768429	p.N1355Kfs*10	c.4065_4068del	pathogenic	38
2768438	p.P1635Qfs*19	c.4904del	likely pathogenic	38
2768457	p.G948Efs*52	c.2838del	likely pathogenic	38
2768460	p.Y655Vfs*18	c.1961dup	pathogenic	38
2768485	p.L1086Dfs*2	c.3254_3255dup	pathogenic	38
2768525	p.K339Rfs*2	c.1016del	pathogenic	38

2768553	p.C1372Efs*2	c.4113_4117del	pathogenic	38
2768554	p.N714Kfs*4	c.2141dup	likely pathogenic	38
2768560	p.H437*	c.1308_1309del	likely pathogenic	38
2768689	p.N1029Kfs*3	c.3087_3088del	likely pathogenic	38
2768761	p.K894Tfs*8	c.2681_2682del	pathogenic	38
2768773	p.V340Gfs*6	c.1016dup	pathogenic	38
2768791	p.Q169*	c.505C>T	pathogenic	38
2768816	p.Y1584Tfs*38	c.4750del	likely pathogenic	38
2768819	p.M1804T	c.5411T>C	likely pathogenic	38
2768830	p.W372Yfs*5	c.1115_1116del	likely pathogenic	38
2768838	p.Q172Nfs*62	c.514del	pathogenic	38
2768840	p.R1443*	c.4327C>T	pathogenic	38
2768848	p.C1718R	c.5152T>C	pathogenic	38
2768851	p.E453Rfs*22	c.1357del	likely pathogenic	38
2768862	p.R1203*	c.3607C>T	pathogenic	38
2768869	p.H1284Tfs*23	c.3850del	likely pathogenic	38
2768880	p.Q1395*	c.4183C>T	pathogenic	38
2802997	p.Q12_V14delinsH	c.36_41del	likely pathogenic	38
2810731	p.R1856*	c.5566C>T	pathogenic	38
2810741	p.E1115*	c.3342_3345del	pathogenic	38
2823388	p.T1249P	c.3745A>C	pathogenic	38
2823406	p.E23Vfs*17	c.68_69del	pathogenic	38
2823407	p.Q1777Pfs*74	c.5329dup	pathogenic	38
2823443	p.E881*	c.2641G>T	pathogenic	38
2823472	p.E1282Afs*26	c.3844_3845insCG	likely pathogenic	38
2823478	p.Q1832R	c.5495A>G	pathogenic	38
2830181	p.R71Kfs*10	c.211dup	pathogenic	38

TCGA: *BRCA1*- negative

Sample ID				
TCGA-A8-A0AD	TCGA-B6-A0WV	TCGA-E2-A155	TCGA-C8-A26V	TCGA-B6-A0IN
TCGA-B6-A0RV	TCGA-C8-A12L	TCGA-AR-A250	TCGA-A8-A09D	TCGA-EW-A1OX
TCGA-AC-A23H	TCGA-AN-A0FW	TCGA-E9-A228	TCGA-S3-AA14	TCGA-EW-A2FV
TCGA-A8-A08L	TCGA-D8-A1X9	TCGA-OL-A5RZ	TCGA-A7-A13D	TCGA-LL-A6FP
TCGA-A8-A09I	TCGA-B6-A0IO	TCGA-C8-A275	TCGA-E9-A226	TCGA-AC-A2B8
TCGA-A2-A0EV	TCGA-BH-A0B1	TCGA-EW-A1OV	TCGA-BH-A1EV	TCGA-BH-A42T
TCGA-AR-A1AP	TCGA-BH-A0H7	TCGA-C8-A1HN	TCGA-A8-A085	TCGA-D8-A27G
TCGA-BH-A1F0	TCGA-C8-A135	TCGA-BH-A0BF	TCGA-OL-A6VO	TCGA-LQ-A4E4
TCGA-A7-A13G	TCGA-E2-A109	TCGA-EW-A1OZ	TCGA-AO-A12C	TCGA-AC-A2FB
TCGA-E2-A14W	TCGA-A2-A0YG	TCGA-BH-A0AW	TCGA-EW-A2FS	TCGA-A2-A0SY
TCGA-BH-A203	TCGA-EW-A1OY	TCGA-AN-A0FY	TCGA-E2-A1LB	TCGA-A8-A09X
TCGA-A1-A0SM	TCGA-B6-A0IK	TCGA-C8-A138	TCGA-E2-A1L7	TCGA-EW-A1J3
TCGA-BH-A0BP	TCGA-BH-A0C0	TCGA-A7-A4SF	TCGA-E9-A1RB	TCGA-EW-A6SC
TCGA-A8-A090	TCGA-D8-A140	TCGA-A2-A0EO	TCGA-A1-A0SJ	TCGA-D8-A27T
TCGA-AC-A5XU	TCGA-D8-A1JG	TCGA-A7-A2KD	TCGA-EW-A1J1	TCGA-A7-A426
TCGA-LD-A9QF	TCGA-BH-A0DX	TCGA-E2-A14T	TCGA-A7-A0D9	TCGA-AC-A6IX
TCGA-A2-A0CU	TCGA-AN-A0AT	TCGA-C8-A130	TCGA-C8-A1HO	TCGA-A2-A4RW
TCGA-BH-A0BW	TCGA-B6-A0I9	TCGA-AN-A04C	TCGA-AO-A03L	TCGA-B6-A0IE
TCGA-E2-A1LE	TCGA-A2-A1FW	TCGA-AR-A254	TCGA-A2-A04X	TCGA-PL-A8LV
TCGA-OL-A5D6	TCGA-AC-A23C	TCGA-BH-A18R	TCGA-AO-A03M	TCGA-AC-A2QH
TCGA-A2-A259	TCGA-EW-A1PD	TCGA-B6-A0IG	TCGA-AR-A0TQ	TCGA-A7-A26G
TCGA-LL-A7T0	TCGA-A8-A08F	TCGA-BH-A0W7	TCGA-C8-A1HF	TCGA-C8-A1HL
TCGA-A8-A09C	TCGA-AN-A0FJ	TCGA-BH-A0E2	TCGA-C8-A273	TCGA-A8-A08J
TCGA-B6-A1KF	TCGA-AO-A0JL	TCGA-A8-A092	TCGA-AO-A0JG	
TCGA-A2-A0CW	TCGA-A2-A4S3	TCGA-BH-A18V	TCGA-AO-A0J5	

ICGC: *BRCA1*- negative

Sample ID							
DO218489	DO227809	DO5801	DO1247	DO3799	DO217934	DO227943	DO224763
DO218488	DO2281	DO4515	DO2575	DO1136	DO1722	DO227944	DO5326
DO218478	DO2275	DO5843	DO44105	DO3388	DO217908	DO227945	DO5312
DO218457	DO227815	DO5836	DO227521	DO2055	DO1727	DO2389	DO224781
DO218428	DO227817	DO4509	DO44103	DO3382	DO1707	DO227947	DO224788
DO218419	DO227820	DO4503	DO44109	DO3376	DO1702	DO227948	DO5305
DO218404	DO2264	DO5829	DO44107	DO2043	DO1767	DO227949	DO224798
DO218408	DO2269	DO5822	DO1237	DO2049	DO1761	DO2383	DO5368
DO218502	DO3590	DO5815	DO1239	DO3370	DO1755	DO227950	DO4036
DO218583	DO227830	DO5884	DO2569	DO4695	DO1743	DO225306	DO4038
DO218560	DO227846	DO3221	DO44101	DO2032	DO1749	DO225305	DO4030
DO218553	DO227854	DO4551	DO2563	DO3364	DO217887	DO225304	DO5361
DO220824	DO227857	DO4557	DO1231	DO2037	DO1732	DO225303	DO4024
DO220826	DO227862	DO3215	DO3892	DO3352	DO1737	DO225302	DO5354
DO220825	DO225207	DO5877	DO1233	DO4683	DO217865	DO225301	DO4012
DO220820	DO225206	DO4545	DO3898	DO3358	DO1792	DO225300	DO4018
DO220822	DO225205	DO5870	DO1235	DO4689	DO1797	DO225309	DO5347
DO220821	DO225203	DO3209	DO227529	DO2026	DO1780	DO225308	DO5340
DO220828	DO225202	DO5863	DO227535	DO2090	DO1786	DO225307	DO5333
DO220827	DO225201	DO3200	DO1227	DO2096	DO1773	DO225317	DO4006
DO218621	DO227860	DO3204	DO1229	DO2084	DO228502	DO225316	DO4000
DO218611	DO225200	DO4533	DO2557	DO2078	DO228506	DO225315	DO4080
DO218605	DO225208	DO4527	DO2551	DO2072	DO228509	DO225314	DO4074
DO218698	DO225177	DO5857	DO1221	DO2067	DO228516	DO225312	DO4068
DO218684	DO225176	DO4521	DO3880	DO3394	DO228521	DO225311	DO4062
DO218656	DO225175	DO5850	DO1223	DO2061	DO1402	DO225310	DO224700
DO218651	DO225174	DO3269	DO3886	DO4713	DO1406	DO225319	DO224709
DO218742	DO225173	DO4599	DO1225	DO4719	DO2737	DO225320	DO5389
DO218736	DO225172	DO3263	DO1215	DO4707	DO2731	DO225327	DO4056
DO218719	DO225171	DO4593	DO1217	DO4701	DO2725	DO225326	DO5382
DO229483	DO225170	DO4587	DO1219	DO225331	DO2719	DO225325	DO4050
DO218796	DO4914	DO3257	DO1211	DO225330	DO2713	DO225324	DO224717
DO218770	DO225179	DO3251	DO2545	DO225339	DO2712	DO225323	DO224713
DO1808	DO225178	DO4581	DO1213	DO225338	DO2700	DO225321	DO4044
DO228283	DO225180	DO4575	DO3874	DO225336	DO1449	DO1098	DO5375
DO1802	DO225188	DO3245	DO1284	DO225333	DO2777	DO225329	DO224722

DO228290	DO225187	DO3239	DO1287	DO225332	DO1440	DO1090	DO224728
DO228293	DO225186	DO4563	DO1286	DO3424	DO2770	DO1092	DO224732
DO228295	DO225185	DO4569	DO1289	DO4756	DO2776	DO1094	DO224730
DO1842	DO225183	DO5898	DO1288	DO225342	DO1445	DO1096	DO224737
DO1848	DO225182	DO5891	DO1281	DO225341	DO1436	DO227903	DO224741
DO1836	DO225181	DO3299	DO1280	DO4749	DO2761	DO227909	DO224746
DO1830	DO4907	DO3293	DO1283	DO225344	DO2764	DO225298	DO4092
DO1820	DO225189	DO3287	DO1282	DO4743	DO1434	DO225297	DO4098
DO1826	DO225191	DO3281	DO227561	DO3412	DO2755	DO3709	DO224755
DO1814	DO225190	DO3275	DO1274	DO225353	DO1429	DO225296	DO4086
DO1885	DO225199	DO5928	DO1273	DO225352	DO217962	DO225295	DO5403
DO1872	DO225198	DO5920	DO1275	DO225351	DO1414	DO225294	DO224887
DO1873	DO225197	DO5912	DO1278	DO225350	DO2749	DO225293	DO224885
DO1866	DO225196	DO5905	DO1277	DO3406	DO1419	DO225292	DO5445
DO1860	DO225194	DO3305	DO1279	DO4737	DO217953	DO225291	DO4116
DO1854	DO3626	DO5960	DO1270	DO225359	DO217952	DO3703	DO4110
DO228247	DO3620	DO4635	DO1272	DO225358	DO2743	DO225299	DO224897
DO228246	DO3614	DO4629	DO1271	DO225356	DO1486	DO3745	DO224895
DO218828	DO4942	DO4623	DO1263	DO225355	DO1481	DO2419	DO224892
DO228256	DO4935	DO4617	DO1262	DO225354	DO1473	DO2413	DO5431
DO218827	DO3602	DO5944	DO1265	DO3400	DO1476	DO2407	DO4104
DO228260	DO3608	DO4611	DO2593	DO4731	DO1468	DO3739	DO5424
DO228262	DO4928	DO5936	DO1264	DO225364	DO2794	DO2401	DO5417
DO228268	DO4921	DO4677	DO1267	DO225363	DO1464	DO3733	DO44273
DO228272	DO1007	DO3346	DO2599	DO225362	DO2796	DO3727	DO44272
DO228276	DO1009	DO4671	DO1266	DO225360	DO2788	DO3721	DO44270
DO1927	DO3668	DO3340	DO1269	DO4725	DO1459	DO3715	DO5486
DO1922	DO1008	DO2008	DO1268	DO225369	DO2783	DO225210	DO4155
DO1926	DO3662	DO2002	DO1260	DO225368	DO1454	DO1128	DO44268
DO1925	DO1000	DO5992	DO1259	DO225367	DO1496	DO225218	DO44260
DO1914	DO4991	DO3334	DO1252	DO225366	DO1497	DO1127	DO44266
DO1908	DO4998	DO4665	DO1251	DO225365	DO1491	DO225217	DO44264
DO1902	DO2335	DO4659	DO1254	DO225375	DO227503	DO225216	DO4149
DO1966	DO1004	DO3328	DO1253	DO225374	DO227507	DO1129	DO5479
DO1960	DO225100	DO5984	DO1256	DO225373	DO2819	DO225214	DO5472
DO1954	DO225108	DO3322	DO1255	DO225372	DO2813	DO225213	DO44258
DO1943	DO2329	DO4647	DO1258	DO225371	DO2808	DO225212	DO44256
DO1948	DO225104	DO3317	DO1257	DO225370	DO2802	DO227871	DO44250
DO1932	DO225103	DO3311	DO2587	DO225379	DO227470	DO225211	DO5465
DO1937	DO225102	DO5976	DO2581	DO225378	DO2854	DO1120	DO4134

DO228328	DO225101	DO4233	DO1250	DO225377	DO1527	DO1121	DO4138
DO228321	DO3650	DO5563	DO1295	DO225376	DO227475	DO3787	DO44248
DO50070	DO3656	DO4239	DO1297	DO3464	DO1522	DO1123	DO44246
DO50072	DO4984	DO5569	DO1299	DO4796	DO227480	DO2455	DO44245
DO1996	DO2323	DO4221	DO1290	DO4794	DO1512	DO225219	DO44243
DO228332	DO225109	DO4227	DO1292	DO2131	DO227483	DO225221	DO4122
DO50064	DO225111	DO5556	DO1291	DO2137	DO1517	DO225220	DO5459
DO50066	DO227776	DO5549	DO1293	DO225386	DO2848	DO2449	DO4128
DO1990	DO2317	DO4215	DO227719	DO225385	DO2842	DO225229	DO5452
DO50060	DO225119	DO4209	DO227730	DO225384	DO1502	DO1116	DO224807
DO50062	DO225118	DO5535	DO227749	DO225383	DO227494	DO225227	DO224805
DO228340	DO4977	DO224560	DO3904	DO225382	DO2836	DO1118	DO4191
DO228345	DO225116	DO224565	DO225056	DO225381	DO2831	DO225226	DO4197
DO50054	DO225115	DO4275	DO225054	DO225380	DO2825	DO225225	DO224811
DO50056	DO225113	DO224577	DO225051	DO225389	DO1569	DO227881	DO224818
DO50058	DO225112	DO5597	DO3946	DO225387	DO2891	DO225223	DO224815
DO1984	DO227773	DO4269	DO225059	DO3454	DO1564	DO225222	DO4185
DO50050	DO4970	DO5590	DO225058	DO2120	DO1550	DO1110	DO224824
DO228350	DO3644	DO4263	DO2617	DO3458	DO2885	DO1112	DO4173
DO228352	DO2311	DO224582	DO2611	DO4787	DO1555	DO2443	DO224833
DO1978	DO225122	DO224588	DO3940	DO2125	DO2879	DO1114	DO4167
DO228356	DO225121	DO224585	DO225067	DO4780	DO1547	DO3775	DO224839
DO50043	DO225120	DO4257	DO225065	DO225397	DO2870	DO225232	DO224835
DO50045	DO227787	DO5583	DO225063	DO225396	DO2874	DO225231	DO4161
DO50047	DO3638	DO4251	DO225061	DO225395	DO1537	DO225230	DO5493
DO50048	DO225129	DO224590	DO3934	DO225394	DO2866	DO1106	DO224844
DO1972	DO225128	DO224598	DO2605	DO225393	DO2860	DO1105	DO224841
DO50049	DO2305	DO5576	DO225070	DO225392	DO1532	DO225239	DO224855
DO50040	DO225127	DO4245	DO225078	DO3442	DO1594	DO1108	DO224860
DO50032	DO225126	DO4299	DO225077	DO4773	DO1599	DO225238	DO224876
DO50034	DO225125	DO4293	DO225076	DO2114	DO1589	DO2437	DO224874
DO50036	DO225124	DO4287	DO225074	DO2108	DO1584	DO225237	DO224879
DO50038	DO225123	DO4281	DO3922	DO4763	DO1579	DO225236	DO44236
DO50030	DO4963	DO5604	DO3928	DO3430	DO1574	DO225235	DO44238
DO50028	DO225133	DO4317	DO225081	DO4766	DO44196	DO227892	DO44230
DO50021	DO225132	DO4311	DO225080	DO3436	DO44194	DO225234	DO44224
DO50023	DO225131	DO5640	DO225089	DO2102	DO44184	DO2431	DO44228
DO50024	DO225139	DO5647	DO225088	DO2174	DO44182	DO1101	DO44222
DO50026	DO227790	DO4305	DO225087	DO2168	DO44188	DO3763	DO44220
DO50017	DO225138	DO5633	DO225086	DO2162	DO1197	DO1103	DO44212

DO228383	DO225137	DO5626	DO225085	DO3494	DO1199	DO225243	DO44218
DO50019	DO225136	DO5625	DO225084	DO2155	DO44192	DO225242	DO44216
DO49027	DO225135	DO5618	DO225082	DO2156	DO44190	DO225241	DO44210
DO49024	DO227795	DO5611	DO3910	DO3488	DO1191	DO225240	DO44208
DO49025	DO2377	DO224643	DO225092	DO3482	DO1193	DO2425	DO44206
DO49022	DO2371	DO224640	DO225091	DO3476	DO1195	DO3757	DO44200
DO49023	DO225144	DO3025	DO225099	DO2143	DO44172	DO225248	DO5528
DO50012	DO225143	DO4353	DO225098	DO2149	DO44178	DO225247	DO5521
DO50013	DO225142	DO4359	DO225097	DO3470	DO44177	DO225246	DO5514
DO50014	DO225141	DO5689	DO225096	DO2198	DO44175	DO225245	DO5500
DO50015	DO225140	DO224647	DO225095	DO2192	DO1185	DO225244	DO5123
DO49021	DO225149	DO5682	DO225093	DO2186	DO1187	DO3751	DO5116
DO228307	DO225148	DO3019	DO2659	DO2180	DO1189	DO225254	DO5109
DO228306	DO225147	DO224654	DO1325	DO4837	DO44180	DO225253	DO5102
DO228300	DO225146	DO224650	DO1328	DO3506	DO1181	DO225252	DO224920
DO228314	DO225145	DO3013	DO1322	DO4830	DO1183	DO225251	DO224928
DO228317	DO2365	DO5675	DO2653	DO3500	DO44168	DO225250	DO224925
DO228319	DO3698	DO4347	DO2647	DO4824	DO44163	DO225259	DO5165
DO228310	DO3692	DO224658	DO3976	DO4817	DO44161	DO225258	DO224930
DO217826	DO225155	DO4341	DO1316	DO4803	DO44166	DO225257	DO5158
DO217800	DO225154	DO224667	DO1319	DO4879	DO44165	DO225255	DO224938
DO1608	DO225153	DO224662	DO3970	DO2216	DO1175	DO1164	DO224934
DO2931	DO225152	DO3001	DO1310	DO3548	DO1174	DO2497	DO5151
DO2937	DO225151	DO5668	DO1313	DO2210	DO1177	DO1166	DO224942
DO1604	DO225150	DO3007	DO2641	DO4872	DO1179	DO1168	DO224948
DO2925	DO2359	DO4335	DO1303	DO3542	DO44170	DO1160	DO224944
DO2919	DO225159	DO5661	DO2635	DO3536	DO1170	DO1162	DO224950
DO2913	DO225158	DO224670	DO1305	DO3530	DO1172	DO2491	DO5137
DO2909	DO225157	DO4329	DO1307	DO4861	DO44159	DO225265	DO224959
DO2903	DO225156	DO5654	DO3964	DO4866	DO44155	DO225264	DO224957
DO228449	DO2353	DO4323	DO1301	DO2204	DO44153	DO225263	DO3070
DO2978	DO3680	DO224680	DO2623	DO3524	DO44148	DO2341	DO3076
DO1648	DO225166	DO224687	DO2629	DO4854	DO44146	DO1014	DO224602
DO228441	DO225165	DO224684	DO3958	DO4847	DO44141	DO2347	DO224609
DO217788	DO225164	DO3067	DO3952	DO3518	DO44140	DO1016	DO224618
DO217786	DO225163	DO3061	DO2694	DO4844	DO228477	DO1015	DO224614
DO217787	DO225162	DO4395	DO1364	DO3512	DO1688	DO1087	DO224625
DO1643	DO225161	DO224693	DO1368	DO2252	DO228483	DO1088	DO224634
DO2972	DO225160	DO224697	DO1360	DO3584	DO228488	DO227916	DO5724
DO2966	DO1018	DO3055	DO1351	DO2258	DO1683	DO1080	DO5717

DO1633	DO1017	DO4383	DO2682	DO3572	DO1678	DO1083	DO5710
DO1638	DO225169	DO4377	DO1354	DO3578	DO228499	DO1082	DO5703
DO228453	DO225168	DO3049	DO2688	DO2246	DO1668	DO1085	DO3107
DO228452	DO225167	DO3043	DO1357	DO2240	DO1663	DO227921	DO3101
DO228455	DO1010	DO5696	DO1348	DO4893	DO2995	DO227924	DO4431
DO2961	DO3674	DO4365	DO227651	DO2234	DO2989	DO1075	DO4437
DO1623	DO1012	DO3037	DO1340	DO3566	DO1658	DO1078	DO5766
DO2955	DO227940	DO3031	DO1342	DO2228	DO2983	DO1071	DO5759
DO228460	DO227941	DO3095	DO2671	DO2222	DO1653	DO1073	DO5752
DO1628	DO227942	DO3089	DO2676	DO3554	DO1693	DO227932	DO4425
DO228479	DO3152	DO3082	DO1345	DO3176	DO1697	DO227935	DO4419
DO228471	DO3194	DO3083	DO225001	DO3170	DO228404	DO2395	DO5745
DO2943	DO5808	DO4407	DO225009	DO4497	DO228409	DO1069	DO4413
DO1613	DO44143	DO5731	DO225007	DO51224	DO228423	DO227936	DO5738
DO2949	DO44138	DO4401	DO225004	DO52556	DO228426	DO227939	DO5214
DO1618	DO44137	DO6112	DO225049	DO51225	DO228427	DO219635	DO5284
DO228433	DO44135	DO6104	DO1372	DO52557	DO228439	DO219639	DO5277
DO217939	DO44133	DO6177	DO1376	DO52547	DO228431	DO219638	DO5270
DO217931	DO44131	DO6160	DO225287	DO51216	DO228430	DO219633	DO5263
DO2293	DO227618	DO6152	DO225286	DO52548	DO225261	DO219631	DO5256
DO2287	DO44127	DO6144	DO225285	DO51217	DO225260	DO219625	DO5291
DO227806	DO44125	DO6195	DO225284	DO52549	DO225269	DO219623	DO5298
DO5005	DO44129	DO6186	DO225283	DO51218	DO225268	DO219629	DO6213
DO5046	DO44121	DO218072	DO225282	DO51219	DO225267	DO219627	DO6204
DO5039	DO227629	DO218062	DO225281	DO52540	DO225266	DO219621	DO6240
DO5032	DO3822	DO218060	DO225280	DO51210	DO1153	DO219615	DO4491
DO5025	DO227593	DO218059	DO1138	DO52542	DO2485	DO219613	DO3164
DO5018	DO3828	DO218182	DO225289	DO51211	DO1155	DO219619	DO4485
DO5012	DO3816	DO218176	DO225288	DO52543	DO1156	DO219617	DO3158
DO5088	DO3810	DO218174	DO1131	DO51212	DO1158	DO219611	DO3146
DO5081	DO3804	DO218173	DO2461	DO52544	DO1151	DO1331	DO4479
DO5060	DO1205	DO218168	DO1132	DO51213	DO1150	DO2665	DO4473
DO5067	DO3868	DO218167	DO3793	DO52545	DO225276	DO1334	DO3140
DO5053	DO1207	DO218159	DO2467	DO51214	DO225275	DO225019	DO3134
DO5095	DO1209	DO218150	DO1134	DO52546	DO225274	DO225017	DO5794
DO223927	DO2539	DO218121	DO6231	DO51215	DO225273	DO225015	DO4467
DO223930	DO1201	DO218279	DO224955	DO51206	DO225272	DO225013	DO4461
DO223937	DO2533	DO219595	DO224965	DO52538	DO225271	DO225022	DO4455
DO223936	DO1200	DO219594	DO224963	DO52539	DO225270	DO225025	DO3122
DO223935	DO1203	DO219593	DO224961	DO51208	DO225279	DO1396	DO3128

DO223934	DO3862	DO218269	DO224967	DO51209	DO225278	DO1398	DO5787
DO223933	DO2527	DO219599	DO224975	DO6136	DO225277	DO1392	DO5780
DO223932	DO3856	DO219597	DO224973	DO6128	DO1142	DO225034	DO4449
DO6000	DO3852	DO219592	DO224971	DO224914	DO1144	DO225032	DO4443
DO6056	DO2521	DO219591	DO224978	DO224911	DO2473	DO225030	DO5773
DO6048	DO2515	DO219590	DO224977	DO5200	DO1146	DO225039	DO3110
DO6040	DO3846	DO218254	DO5193	DO5207	DO2479	DO225037	DO3116
DO6032	DO3840	DO219589	DO224987	DO5249	DO1148	DO225035	DO3188
DO6024	DO2503	DO219588	DO224985	DO5242	DO1140	DO1384	DO3182
DO6096	DO3834	DO218205	DO224982	DO5235	DO219647	DO1388	DO51229
DO6088	DO2509	DO218384	DO224989	DO5228	DO219646	DO1380	DO44113
DO6080	DO227510	DO218388	DO5186	DO5221	DO219645	DO225044	DO218333
DO6072	DO44115	DO218377	DO224990	DO1241	DO219640	DO52551	DO51222
DO6064	DO52555	DO219609	DO224996	DO1243	DO219644	DO51220	DO52554
DO52561	DO51227	DO219608	DO224995	DO1245	DO219642	DO52552	DO51223
DO51230	DO52559	DO219607	DO224992	DO6222	DO219641	DO51221	DO44117
DO51231	DO51228	DO219605	DO5179	DO227517	DO219637	DO224900	DO224904
DO52560	DO225262	DO218347	DO224999	DO52558	DO219636	DO44111	DO1249
DO5172	DO52553						

Cosmic: *BRCA1*- negative

Sample ID							
1235092	2724744	2768283	2807311	2830157	2717669	2767937	2768748
1235093	2724758	2768284	2807312	2830159	2717678	2767938	2768749
1235094	2724769	2768285	2807313	2830162	2717687	2767939	2768757
1235096	2724823	2768286	2807314	2830166	2717714	2767940	2768758
1235097	2724877	2768288	2807316	2830171	2717739	2767941	2768759
1235099	2724888	2768289	2807317	2830173	2717855	2767942	2768760
1235101	2724918	2768290	2807318	2830174	2717860	2767943	2768762
1235102	2724962	2768291	2807319	2830177	2717938	2767945	2768763
1235106	2724969	2768292	2807320	2830178	2717944	2767946	2768764
1235108	2725000	2768293	2807321	2830182	2717988	2767947	2768765
1235110	2725003	2768294	2807322	2830183	2718174	2767948	2768766
1317049	2725028	2768295	2807323	2830184	2718199	2767949	2768767
1446136	2725115	2768296	2807324	2830185	2718223	2767950	2768769
1446139	2725156	2768297	2807325	2830189	2718255	2767951	2768770
1446145	2725157	2768298	2810709	2830193	2718284	2767952	2768771
1446151	2725191	2768299	2810710	2830197	2718330	2767953	2768772
1520432	2725271	2768300	2810711	2830199	2718442	2767954	2768774
1520442	2725275	2768301	2810712	2830200	2718520	2767955	2768776

1520443	2725299	2768302	2810713	2830201	2718521	2767956	2768777
1520444	2725357	2768303	2810714	2830210	2718531	2767957	2768778
1520445	2725368	2768304	2810715	2830212	2718535	2767958	2768779
1520446	2725387	2768305	2810716	2830217	2718536	2767959	2768780
1520447	2725406	2768306	2810717	2830219	2718537	2767960	2768781
1520448	2725414	2768307	2810718	2830224	2718539	2767961	2768782
1520449	2725438	2768308	2810719	2830225	2718643	2767962	2768783
1520450	2725474	2768309	2810720	2830226	2718646	2767964	2768784
1520451	2725489	2768310	2810721	2830232	2718657	2767965	2768786
1520452	2725520	2768311	2810722	2830236	2718661	2767966	2768787
1520453	2725523	2768312	2810723	2830237	2718665	2767967	2768790
1520454	2725525	2768313	2810724	2830238	2718676	2767968	2768792
1520455	2725529	2768314	2810725	2830239	2718718	2767969	2768794
1520456	2725534	2768315	2810726	2830240	2718720	2767970	2768795
1520457	2725596	2768316	2810727	2830242	2718746	2767971	2768796
1520458	2725600	2768317	2810728	2830251	2718780	2767972	2768797
1520459	2725601	2768318	2810729	2830254	2718826	2767973	2768798
1520460	2725614	2768319	2810730	2830255	2718887	2767974	2768799
1520462	2725622	2768320	2810732	2830256	2718895	2767975	2768800
1520463	2725624	2768321	2810733	2830258	2718903	2767976	2768801
1520465	2725628	2768322	2810734	2830260	2718913	2767978	2768802
1520466	2725642	2768323	2810735	2830261	2718941	2767979	2768803
1520467	2725653	2768324	2810736	2830262	2718945	2767980	2768804
1520468	2725659	2768325	2810737	2830268	2718990	2767981	2768805
1520469	2725666	2768326	2810738	2830270	2718994	2767982	2768806
1520471	2725679	2768327	2810739	2830276	2719056	2767983	2768807
1520473	2725758	2768328	2810740	2830277	2719074	2767984	2768808
1520474	2725794	2768329	2810742	2830279	2719083	2767985	2768809
1520476	2725821	2768330	2810743	2830280	2719158	2767986	2768810
1520477	2725829	2768331	2810744	2830285	2719166	2767987	2768811
1520481	2725870	2768332	2810745	2830288	2719184	2767988	2768812
1520483	2725889	2768333	2810746	2830289	2719211	2767989	2768813
1520486	2725892	2768334	2810747	2830290	2719234	2767990	2768814
1520488	2725896	2768335	2810748	2830298	2719276	2767991	2768815
1520491	2726006	2768336	2810749	2830299	2719280	2767992	2768817
1520492	2726024	2768337	2810750	2830301	2719294	2767993	2768818
1520493	2726093	2768338	2810751	2830302	2719295	2767994	2768820
1520494	2726117	2768339	2810752	2830304	2719321	2767995	2768822
1520495	2726129	2768340	2810753	2830305	2719322	2767996	2768825
1520496	2726191	2768342	2810754	2830310	2719323	2767997	2768826
1520497	2726195	2768343	2810755	2830311	2719324	2767998	2768827

1520498	2726197	2768344	2810756	2830313	2719331	2767999	2768828
1520499	2726244	2768345	2810757	2830319	2719356	2768000	2768829
1520500	2726262	2768346	2810758	2830321	2719429	2768001	2768831
1520501	2726326	2768347	2810759	2830323	2719443	2768002	2768832
1520502	2726356	2768349	2810760	2830324	2719480	2768003	2768833
1520503	2726390	2768350	2810761	2830325	2719482	2768004	2768834
1520504	2726400	2768351	2810762	2830326	2719535	2768005	2768835
1520506	2726409	2768353	2810763	2830329	2719613	2768006	2768836
1520507	2726426	2768354	2810764	2830332	2719627	2768008	2768841
1520508	2726435	2768356	2810765	2830337	2719628	2768009	2768842
1520509	2726475	2768357	2810766	2830350	2719636	2768010	2768843
1520510	2726477	2768359	2810767	2830351	2719659	2768012	2768844
1520511	2726511	2768360	2810768	2830352	2719672	2768013	2768845
1520513	2726517	2768361	2810769	2830353	2719673	2768014	2768846
1520514	2726521	2768362	2811671	2830354	2719689	2768015	2768847
1520517	2726538	2768363	2811672	2830355	2719718	2768016	2768849
1520519	2726562	2768364	2811673	2830357	2719737	2768017	2768850
1520520	2726584	2768365	2811674	2830358	2719777	2768018	2768852
1520521	2726586	2768366	2811675	2830359	2719833	2768019	2768853
1520523	2726622	2768367	2811676	2830360	2719866	2768020	2768854
1520525	2726678	2768370	2811677	2830362	2719905	2768021	2768855
1520526	2726687	2768371	2811678	2830364	2719959	2768022	2768859
1520527	2726690	2768372	2811679	2830367	2719960	2768023	2768860
1520528	2726691	2768373	2811680	2830368	2719988	2768024	2768861
1520529	2726793	2768374	2811681	2830377	2720037	2768025	2768863
1520530	2726842	2768375	2811682	2830378	2720075	2768026	2768864
1520531	2726861	2768377	2811683	2830381	2720118	2768027	2768865
1520533	2726944	2768378	2811685	2830383	2720133	2768028	2768867
1520536	2726951	2768379	2811686	2830384	2720135	2768029	2768868
1520537	2726997	2768380	2811687	2830387	2720138	2768030	2768870
1520538	2727049	2768381	2811688	2830391	2720147	2768031	2768871
1520540	2727057	2768382	2811691	2830393	2720251	2768032	2768872
1520543	2727074	2768383	2811692	2830397	2720252	2768034	2768873
1520556	2727140	2768384	2811693	2830400	2720270	2768035	2768874
1520557	2727157	2768385	2811694	2830404	2720339	2768036	2768875
1520558	2727159	2768386	2811695	2830405	2720360	2768037	2768876
1520559	2727166	2768387	2811696	2830406	2720376	2768038	2768877
1520560	2727167	2768388	2811697	2830407	2720424	2768039	2768878
1520561	2727223	2768389	2811698	2830408	2720492	2768041	2768879
1520562	2727256	2768390	2811699	2830412	2720523	2768043	2802968
1520563	2727262	2768392	2811700	2830414	2720537	2768044	2802969

1520564	2727269	2768394	2811701	2830415	2720600	2768045	2802971
1520565	2727281	2768395	2811702	2830417	2720603	2768046	2802973
1520566	2727291	2768398	2811703	2830425	2720617	2768047	2802975
1520567	2727302	2768399	2811704	2830426	2720688	2768048	2802976
1520568	2727323	2768400	2811705	2830427	2720718	2768050	2802977
1520569	2727394	2768401	2811706	2830428	2720724	2768051	2802978
1520570	2727400	2768402	2811707	2830429	2720754	2768052	2802979
1520571	2727430	2768403	2811708	2830430	2720761	2768053	2802980
1520572	2727452	2768404	2811709	2830431	2720792	2768054	2802981
1520573	2727466	2768405	2811710	2830433	2720795	2768055	2802982
1520574	2767617	2768408	2811711	2830434	2720797	2768056	2802983
1520575	2767630	2768410	2811713	2830435	2720865	2768057	2802984
1520576	2767631	2768411	2811714	2830436	2720871	2768058	2802986
1520577	2767633	2768412	2811717	2830437	2720885	2768059	2802987
1520578	2767634	2768413	2811718	2830438	2720941	2768060	2802988
1520579	2767635	2768414	2811719	2830439	2720963	2768061	2802989
1520580	2767636	2768416	2811720	2830443	2720966	2768062	2802991
1520581	2767637	2768430	2811721	2830446	2721053	2768063	2802992
1520582	2767638	2768432	2811722	2830448	2721065	2768065	2802995
1520583	2767639	2768433	2811723	2830451	2721068	2768068	2802998
1520584	2767640	2768434	2811724	2830452	2721082	2768070	2803000
1520585	2767641	2768435	2811725	2830454	2721153	2768071	2803001
1520587	2767643	2768436	2811726	2830456	2721210	2768072	2803003
1520589	2767644	2768437	2811727	2830459	2721244	2768073	2803004
1520592	2767645	2768439	2811728	2830460	2721263	2768074	2803005
1520594	2767646	2768440	2811729	2830463	2721292	2768075	2803006
1520595	2767647	2768441	2811730	2830464	2721297	2768076	2803007
1520596	2767648	2768442	2811731	2830465	2721322	2768077	2803010
1520598	2767649	2768443	2811732	2830469	2721327	2768078	2803012
1520599	2767650	2768444	2811733	2830471	2721330	2768079	2803014
1520600	2767651	2768445	2811734	2830472	2721348	2768080	2803016
1520601	2767654	2768446	2811735	2830473	2721360	2768081	2803017
1520602	2767655	2768447	2811736	2830480	2721376	2768082	2803019
1520603	2767656	2768448	2811737	2830484	2721391	2768083	2803020
1520604	2767657	2768449	2811738	2830485	2721409	2768084	2803021
1520605	2767658	2768450	2811739	2830486	2721413	2768085	2803022
1520608	2767659	2768451	2811740	2830487	2721414	2768086	2803023
1520614	2767660	2768452	2811741	2830489	2721415	2768088	2803025
1749320	2767661	2768453	2811743	2830492	2721420	2768089	2807193
1775596	2767662	2768454	2811744	2830495	2721476	2768090	2807194
1775599	2767663	2768456	2812134	2830497	2721483	2768093	2807195

1775602	2767664	2768458	2812135	2830499	2721524	2768094	2807196
1775608	2767665	2768459	2812136	2830500	2721562	2768095	2807197
1914128	2767666	2768461	2821971	2830502	2721572	2768096	2807199
1914136	2767667	2768462	2821973	2830503	2721580	2768097	2807200
1914144	2767668	2768463	2821974	2830504	2721609	2768098	2807201
1914156	2767669	2768464	2821976	2830505	2721662	2768099	2807202
1914168	2767670	2768465	2821978	2830507	2721693	2768100	2807203
1914178	2767671	2768466	2821979	2830510	2721716	2768101	2807204
1914198	2767672	2768467	2821980	2830512	2721755	2768102	2807205
1914214	2767673	2768468	2821981	2830513	2721772	2768103	2807206
1914228	2767674	2768469	2821982	2830514	2721797	2768104	2807207
1914238	2767675	2768470	2821983	2830517	2721801	2768105	2807208
1914248	2767676	2768471	2821984	2830519	2721808	2768106	2807209
1914266	2767678	2768472	2821985	2830520	2721860	2768107	2807210
1914294	2767679	2768473	2821986	2830525	2721864	2768108	2807211
1914306	2767680	2768474	2821988	2830529	2721919	2768109	2807212
1914320	2767681	2768475	2821989	2830530	2721997	2768110	2807213
1914328	2767682	2768476	2821990	2830531	2722006	2768111	2807214
1914340	2767683	2768477	2821991	2830532	2722016	2768112	2807215
1914350	2767684	2768478	2821992	2830534	2722061	2768113	2807216
1914366	2767685	2768479	2821993	2830536	2722073	2768114	2807217
1914408	2767686	2768480	2821994	2830539	2722096	2768115	2807218
1914428	2767687	2768481	2821995	2830542	2722101	2768116	2807219
1914438	2767688	2768482	2821996	2830544	2722170	2768118	2807220
1914450	2767689	2768484	2821997	2830546	2722201	2768120	2807221
1914458	2767690	2768486	2822001	2830547	2722217	2768127	2807223
1914488	2767691	2768487	2822002	2830548	2722244	2768128	2807224
1914504	2767692	2768488	2822004	2830549	2722263	2768129	2807225
1971251	2767693	2768489	2822005	2830550	2722290	2768130	2807226
1971252	2767696	2768490	2822006	2830551	2722314	2768132	2807227
1971254	2767697	2768491	2822007	2830552	2722357	2768133	2807228
1971258	2767698	2768492	2822008	2830554	2722447	2768134	2807229
1971259	2767699	2768493	2822010	2830555	2722448	2768135	2807230
1971263	2767700	2768494	2822011	2830556	2722464	2768136	2807231
1971265	2767701	2768495	2822012	2830557	2722467	2768137	2807232
1971266	2767702	2768496	2822013	2830560	2722469	2768139	2807233
1971270	2767704	2768497	2822015	2830563	2722517	2768140	2807234
1971271	2767705	2768500	2822016	2830564	2722548	2768141	2807235
2234058	2767707	2768501	2822017	2830565	2722629	2768142	2807236
2318498	2767708	2768502	2822018	2830566	2722630	2768143	2807237
2318499	2767709	2768503	2822019	2830567	2722685	2768144	2807238

2549272	2767711	2768504	2822020	2830568	2722725	2768145	2807239
2579111	2767712	2768505	2822021	2830569	2722765	2768146	2807240
2579112	2767713	2768507	2822022	2830570	2722812	2768147	2807241
2579115	2767715	2768508	2822023	2830571	2722833	2768148	2807242
2579119	2767716	2768509	2822024	2830572	2722835	2768149	2807243
2579126	2767717	2768510	2822025	2830574	2722873	2768150	2807244
2657323	2767718	2768511	2822029	2830575	2722895	2768151	2807245
2657325	2767719	2768513	2822030	2830576	2722909	2768152	2807246
2657326	2767720	2768514	2822031	2830577	2722934	2768155	2807248
2657327	2767721	2768517	2822032	2830578	2722946	2768156	2807249
2657328	2767722	2768518	2822033	2830579	2723002	2768157	2807250
2657329	2767724	2768519	2822036	2830580	2723010	2768158	2807251
2657330	2767725	2768520	2822037	2830581	2723151	2768159	2807252
2657331	2767727	2768522	2822038	2830582	2723198	2768160	2807253
2657332	2767728	2768523	2822039	2830583	2723208	2768161	2807254
2657333	2767729	2768524	2822040	2830585	2723227	2768162	2807255
2657334	2767730	2768526	2822041	2830586	2723287	2768163	2807256
2657335	2767732	2768528	2822042	2830587	2723289	2768164	2807257
2657336	2767734	2768529	2822043	2830588	2723309	2768165	2807259
2689711	2767735	2768530	2822044	2830589	2723318	2768166	2807261
2689713	2767736	2768531	2822045	2830590	2723320	2768167	2807262
2689714	2767737	2768532	2822046	2830591	2723365	2768168	2807263
2689716	2767738	2768533	2822047	2830592	2723389	2768169	2807264
2689717	2767739	2768535	2822048	2830594	2723446	2768170	2807265
2689718	2767740	2768536	2822049	2830595	2723473	2768173	2807266
2689719	2767741	2768537	2822050	2830596	2723626	2768175	2807267
2689720	2767742	2768538	2822051	2830597	2723665	2768176	2807268
2689721	2767743	2768539	2822052	2830598	2723673	2768177	2807269
2689722	2767744	2768540	2822053	2830599	2723687	2768178	2807270
2689723	2767745	2768541	2822054	2830600	2723700	2768179	2807271
2689724	2767746	2768542	2822055	2830601	2723781	2768181	2807272
2689725	2767747	2768543	2822056	2830602	2723805	2768182	2807273
2689726	2767749	2768544	2822057	2830603	2723826	2768183	2807274
2689727	2767750	2768546	2822058	2830604	2723852	2768184	2807275
2690554	2767751	2768547	2822059	2830609	2723858	2768185	2807276
2690555	2767752	2768548	2822061	2830610	2723864	2768186	2807277
2690556	2767753	2768549	2822114	2830611	2723886	2768187	2807278
2690557	2767754	2768551	2823385	2830612	2723957	2768188	2807279
2690559	2767756	2768555	2823386	2830613	2723958	2768189	2807280
2690560	2767757	2768556	2823387	2830614	2723959	2768190	2807281
2690561	2767758	2768557	2823389	2830621	2723974	2768191	2807282

2690562	2767759	2768558	2823390	2830622	2723994	2768199	2807283
2690563	2767760	2768559	2823391	2830623	2724002	2768254	2807284
2690564	2767761	2768561	2823392	2830625	2724113	2768255	2807285
2690565	2767762	2768562	2823393	2830626	2724168	2768256	2807286
2690567	2767763	2768563	2823394	2830627	2724171	2768257	2807287
2690568	2767764	2768566	2823395	2830629	2724172	2768258	2807288
2690569	2767765	2768567	2823396	2830630	2724192	2768259	2807289
2690570	2767766	2768568	2823397	2830631	2724209	2768260	2807290
2690571	2767767	2768569	2823399	2830632	2724234	2768261	2807291
2690572	2767768	2768570	2823400	2830633	2724282	2768262	2807292
2690573	2767769	2768571	2823401	2830636	2724309	2768263	2807293
2690574	2767771	2768572	2823402	2830637	2724313	2768264	2807294
2690575	2767772	2768573	2823403	2830638	2724320	2768265	2807295
2690576	2767773	2768574	2823404	2830639	2724338	2768266	2807296
2690577	2767774	2768575	2823405	2830640	2724369	2768267	2807297
2690578	2767775	2768576	2823408	2830641	2724474	2768269	2807298
2690579	2767776	2768577	2823409	2830642	2724490	2768270	2807299
2690581	2767778	2768578	2823410	2830646	2724495	2768271	2807300
2690582	2767779	2768579	2823411	2830647	2724540	2768272	2807301
2690584	2767780	2768580	2823412	2830648	2724566	2768273	2807302
2690585	2767781	2768581	2823413	2830649	2724587	2768274	2807303
2690586	2767782	2768582	2823414	2830650	2724592	2768275	2807304
2690587	2767783	2768583	2823416	2830651	2724595	2768277	2807305
2690588	2767784	2768584	2823417	2830652	2724596	2768278	2807306
2690590	2767785	2768585	2823418	2830653	2724631	2768279	2807307
2690591	2767786	2768586	2823419	2830659	2724655	2768280	2807308
2690592	2767787	2768587	2823420	2830660	2724656	2768281	2807309
2690593	2767788	2768590	2823421	2830661	2724696	2768282	2807310
2690594	2767789	2768591	2823422	2830662	2829325	2830868	2829868
2690595	2767790	2768592	2823423	2830665	2829328	2830869	2829872
2690596	2767791	2768593	2823424	2830666	2829329	2830870	2829874
2690597	2767792	2768594	2823425	2830667	2829330	2830872	2829876
2690598	2767793	2768595	2823426	2830668	2829331	2830874	2829878
2690599	2767794	2768596	2823427	2830669	2829332	2830875	2829880
2690600	2767795	2768597	2823428	2830670	2829333	2830876	2829886
2690601	2767796	2768598	2823429	2830671	2829334	2830877	2829887
2690602	2767797	2768599	2823430	2830673	2829340	2830878	2829890
2690603	2767798	2768600	2823431	2830674	2829347	2830879	2829891
2690604	2767799	2768601	2823432	2830675	2829349	2830880	2829893
2690605	2767800	2768602	2823433	2830676	2829356	2830882	2829895
2690606	2767801	2768603	2823434	2830677	2829357	2830883	2829899

2690607	2767802	2768604	2823435	2830679	2829358	2830884	2829900
2690608	2767803	2768605	2823436	2830680	2829363	2830885	2829902
2690609	2767804	2768606	2823437	2830681	2829364	2830886	2829903
2690611	2767805	2768607	2823438	2830682	2829365	2830887	2829908
2690612	2767806	2768608	2823439	2830683	2829369	2830888	2829912
2690613	2767807	2768609	2823440	2830684	2829373	2830889	2829914
2690614	2767808	2768610	2823441	2830685	2829377	2830890	2829916
2690615	2767809	2768611	2823442	2830686	2829378	2830891	2829917
2690616	2767810	2768612	2823444	2830687	2829381	2830892	2829920
2690617	2767811	2768613	2823445	2830688	2829382	2830893	2829922
2690620	2767812	2768614	2823446	2830691	2829385	2830894	2829927
2690621	2767813	2768615	2823447	2830693	2829397	2830895	2829928
2690622	2767814	2768616	2823448	2830694	2829400	2830896	2829930
2690623	2767815	2768617	2823449	2830695	2829403	2830897	2829931
2690624	2767816	2768618	2823450	2830696	2829412	2830898	2829933
2690625	2767817	2768619	2823451	2830697	2829418	2830899	2829934
2690626	2767818	2768620	2823452	2830698	2829423	2830900	2829936
2690627	2767819	2768621	2823453	2830702	2829435	2830901	2829937
2690628	2767820	2768622	2823454	2830703	2829438	2830902	2829944
2690629	2767821	2768623	2823455	2830708	2829446	2830903	2829951
2690630	2767822	2768624	2823456	2830709	2829449	2830904	2829952
2690631	2767823	2768625	2823457	2830713	2829452	2830905	2829961
2690632	2767824	2768626	2823458	2830714	2829453	2830906	2829965
2690633	2767825	2768627	2823459	2830715	2829454	2830907	2829970
2690634	2767826	2768628	2823460	2830716	2829462	2830908	2829972
2690636	2767827	2768629	2823461	2830717	2829467	2830909	2829976
2690637	2767828	2768630	2823462	2830718	2829468	2830911	2829977
2690638	2767829	2768631	2823463	2830719	2829470	2830912	2829981
2690639	2767830	2768632	2823464	2830720	2829472	2830913	2829983
2690640	2767831	2768633	2823465	2830721	2829474	2830914	2829985
2690641	2767832	2768634	2823466	2830722	2829475	2830915	2829989
2690643	2767833	2768635	2823467	2830724	2829479	2830917	2829990
2690646	2767834	2768636	2823468	2830725	2829489	2830918	2829993
2690647	2767835	2768637	2823469	2830727	2829503	2830919	2829996
2690648	2767836	2768638	2823470	2830728	2829504	2830920	2829997
2690649	2767837	2768639	2823471	2830731	2829507	2830921	2830009
2690650	2767838	2768640	2823473	2830734	2829512	2830922	2830013
2714466	2767839	2768641	2823474	2830735	2829513	2830923	2830015
2714495	2767840	2768642	2823475	2830737	2829516	2830924	2830018
2714510	2767841	2768643	2823476	2830740	2829517	2830925	2830023
2714562	2767842	2768644	2823477	2830741	2829519	2830926	2830027

2714583	2767843	2768645	2823479	2830742	2829527	2830927	2830028
2714592	2767844	2768646	2823480	2830743	2829531	2830928	2830031
2714610	2767845	2768647	2823481	2830744	2829533	2830929	2830036
2714639	2767846	2768648	2823482	2830748	2829539	2830930	2830042
2714651	2767847	2768649	2823483	2830749	2829540	2830931	2830046
2714656	2767848	2768650	2823484	2830751	2829541	2830932	2830058
2714673	2767849	2768651	2823485	2830752	2829546	2830933	2830060
2714710	2767850	2768652	2823486	2830753	2829550	2830934	2830061
2714750	2767851	2768653	2823487	2830754	2829559	2830935	2830064
2714763	2767852	2768654	2823488	2830756	2829560	2830936	2830065
2714775	2767853	2768655	2823489	2830758	2829561	2830937	2830070
2714782	2767854	2768656	2823490	2830759	2829562	2830938	2830072
2714789	2767855	2768657	2823491	2830760	2829565	2830939	2830073
2714791	2767856	2768658	2829082	2830763	2829568	2830940	2830076
2714814	2767857	2768659	2829083	2830764	2829569	2830941	2830077
2714819	2767858	2768660	2829086	2830766	2829572	2830942	2830079
2714892	2767859	2768661	2829094	2830767	2829573	2830943	2830080
2714941	2767860	2768662	2829095	2830768	2829578	2830944	2830083
2714944	2767861	2768663	2829100	2830769	2829584	2830945	2830084
2714946	2767862	2768664	2829104	2830770	2829589	2830946	2830085
2714981	2767863	2768665	2829106	2830771	2829596	2830947	2830086
2714988	2767864	2768666	2829112	2830772	2829601	2830948	2830088
2715022	2767865	2768667	2829118	2830774	2829605	2830949	2830090
2715061	2767866	2768668	2829119	2830775	2829608	2830950	2830094
2715100	2767867	2768669	2829121	2830779	2829609	2830951	2830096
2715106	2767868	2768670	2829122	2830781	2829611	2830954	2830097
2715111	2767869	2768671	2829123	2830782	2829614	2830955	2830098
2715165	2767870	2768672	2829124	2830783	2829618	2830956	2830100
2715232	2767871	2768673	2829128	2830784	2829621	2830957	2830103
2715237	2767872	2768674	2829129	2830786	2829628	2830958	2830106
2715260	2767873	2768675	2829139	2830787	2829631	2830959	2830109
2715269	2767874	2768676	2829147	2830789	2829633	2830961	2830111
2715287	2767875	2768677	2829148	2830790	2829643	2830962	2830112
2715302	2767876	2768679	2829149	2830791	2829644	2830963	2830113
2715340	2767877	2768680	2829150	2830792	2829647	2830964	2830120
2715348	2767878	2768682	2829155	2830795	2829656	2830965	2830122
2715384	2767879	2768683	2829157	2830797	2829659	2830966	2830127
2715398	2767880	2768685	2829158	2830798	2829675	2830967	2830128
2715433	2767881	2768686	2829159	2830800	2829676	2830968	2830129
2715446	2767882	2768687	2829166	2830804	2829679	2830969	2830133
2715469	2767883	2768688	2829167	2830805	2829682	2830970	2830135

2715476	2767884	2768690	2829168	2830806	2829683	2830971	2830137
2715481	2767885	2768691	2829170	2830807	2829684	2830973	2830138
2715568	2767886	2768692	2829177	2830809	2829685	2830975	2830139
2715585	2767887	2768693	2829178	2830810	2829686	2830976	2830140
2715592	2767888	2768694	2829181	2830811	2829690	2830977	2830143
2715642	2767889	2768695	2829182	2830812	2829692	2830978	2830145
2715651	2767890	2768696	2829185	2830813	2829695	2830979	2830146
2715721	2767891	2768697	2829187	2830814	2829710	2830980	2830152
2715727	2767892	2768698	2829189	2830817	2829712	2830981	2903348
2715746	2767893	2768699	2829191	2830819	2829715	2830982	2903350
2715802	2767894	2768700	2829194	2830820	2829716	2830983	2903352
2715971	2767895	2768701	2829200	2830822	2829723	2830984	2903354
2716022	2767896	2768702	2829203	2830823	2829732	2830986	2903358
2716055	2767897	2768703	2829208	2830825	2829735	2830987	2909923
2716075	2767898	2768704	2829211	2830826	2829739	2830989	2909924
2716260	2767899	2768705	2829212	2830827	2829741	2830990	2909925
2716262	2767900	2768706	2829217	2830828	2829743	2830991	2909926
2716318	2767901	2768707	2829222	2830831	2829744	2830992	2909927
2716358	2767902	2768708	2829223	2830832	2829745	2830993	2909928
2716375	2767903	2768709	2829231	2830833	2829754	2830994	
2716391	2767904	2768710	2829232	2830834	2829756	2830995	
2716448	2767905	2768711	2829236	2830835	2829763	2830996	
2716513	2767906	2768713	2829237	2830836	2829767	2830997	
2716620	2767907	2768714	2829238	2830837	2829777	2830998	
2716708	2767908	2768715	2829243	2830838	2829778	2830999	
2716737	2767909	2768716	2829246	2830839	2829779	2903320	
2716757	2767910	2768717	2829249	2830840	2829788	2903321	
2716759	2767911	2768718	2829254	2830841	2829792	2903322	
2716771	2767912	2768719	2829255	2830842	2829796	2903323	
2716821	2767913	2768720	2829260	2830843	2829798	2903324	
2716842	2767914	2768721	2829261	2830844	2829799	2903325	
2717035	2767915	2768723	2829262	2830845	2829801	2903326	
2717089	2767916	2768724	2829265	2830846	2829810	2903327	
2717095	2767917	2768726	2829267	2830847	2829811	2903328	
2717110	2767918	2768727	2829271	2830848	2829812	2903329	
2717129	2767919	2768728	2829273	2830849	2829813	2903330	
2717156	2767920	2768729	2829276	2830850	2829816	2903331	
2717165	2767921	2768730	2829280	2830851	2829819	2903332	
2717250	2767922	2768731	2829283	2830852	2829824	2903333	
2717254	2767923	2768732	2829285	2830853	2829827	2903334	
2717316	2767924	2768733	2829286	2830854	2829828	2903335	

2717327	2767925	2768734	2829287	2830855	2829829	2903336
2717371	2767926	2768735	2829291	2830856	2829830	2903337
2717373	2767927	2768736	2829292	2830857	2829831	2903338
2717375	2767928	2768737	2829299	2830858	2829837	2903339
2717382	2767929	2768738	2829301	2830859	2829838	2903340
2717386	2767930	2768739	2829302	2830860	2829841	2903341
2717422	2767931	2768742	2829303	2830861	2829848	2903342
2717493	2767932	2768743	2829306	2830862	2829850	2903343
2717538	2767933	2768744	2829310	2830863	2829851	2903344
2717559	2767934	2768745	2829314	2830864	2829853	2903345
2717601	2767935	2768746	2829322	2830866	2829860	2903346
2717628	2767936	2768747	2829323	2830867	2829867	2903347

Supplementary Table S3. Median and p-value of non-synonymous variant from Mann-Whitney U test of all databases.

**Type of counts**	***BRCA1*- positive (median)**	**BRCA1- negative (median)**	**p-value (2-tailed)**
TCGA			
Total variant count	83.50 (Q_1 59.75-$Q_3$140.25)	39.00 ($Q_1$22.00-$Q_3$65.00)	<0.001
SNV count	80.00 ($Q_1$53.00-$Q_3$137.25)	36.00 ($Q_1$19.00-$Q_3$56.00)	<0.001
Indel count	5.50 ($Q_1$2.00-$Q_3$9.00)	3.00 ($Q_1$1.00-$Q_3$4.00)	0.019
ICGC			
Total variant count	95.00 ($Q_1$66.00-$Q_3$140.00)	36.00 ($Q_1$23.00-$Q_3$62.00)	<0.001
SNV count	91.00 ($Q_1$63.00-$Q_3$139.00)	34.00 ($Q_1$22.00-$Q_3$60.00)	<0.001
Indel count	2.00 ($Q_1$0.00-$Q_3$6.00)	1.00 ($Q_1$0.00-$Q_3$2.00)	0.032
Cosmic			
Total variant count	6.50 ($Q_1$4.00-$Q_3$8.00)	2.00 ($Q_1$1.00-$Q_3$2.00)	<0.001
SNV count	5.00 ($Q_1$2.50-$Q_3$7.00)	1.00 ($Q_1$1.00-$Q_3$2.00)	<0.001
Indel count	1.00 ($Q_1$0.00-$Q_3$2.00)	0.00 ($Q_1$0.00-$Q_3$1.00)	0.031

Supplementary Table S4. Binding prediction results between epitopes of recurrent somatic mutation and MHC class II.

Somatic mutation	Allele	Peptide	Length	Method	Adjusted percentile rank	IC50	Allele/Haplotype frequency (%)
PIK3CA E542K	-	-	-	-	-	-	-
PIK3CA E545K	HLA-DRB1*07:01	KQEKDFLWSHRHYCV	15	Consensus	8.3	-	12.21
	HLA-DPA1*01:03/DPB1*04:01	KQEKDFLWSHRHYCVTI	17	NetMHCIIpan	44.28	881.39	17.41
	HLA-DPA1*01:03/DPB1*04:01	KQEKDFLWSHRHYCVTIP	18	NetMHCIIpan	85.83	904.49	17.41
	HLA-DPA1*01:03/DPB1*04:01	TKQEKDFLWSHRHYCVTI	18	NetMHCIIpan	82.87	875.65	17.41
PIK3CA H1047R	HLA-DRB1*04:01	ALEYFMKQMNDAHR	14	Consensus	4.85	-	6.37
	HLA-DRB1*04:01	ALEYFMKQMNDAHRG	15	Consensus	5.2	-	6.37
	HLA-DRB3*02:02	ALEYFMKQMNDAHRG	15	NetMHCIIpan	14	850.37	16.40
	HLA-DRB1*04:01	ALEYFMKQMNDAHRGG	16	Consensus	6.93	-	6.37
	HLA-DRB3*02:02	ALEYFMKQMNDAHRGG	16	NetMHCIIpan	17.31	936.09	16.40
	HLA-DRB1*04:01	EALEYFMKQMNDAHR	15	Consensus	5.2	-	6.37
	HLA-DRB1*04:01	EALEYFMKQMNDAHRG	16	Consensus	6.93	-	6.37
	HLA-DRB3*02:02	EALEYFMKQMNDAHRG	16	NetMHCIIpan	18.47	989.67	16.40
	HLA-DPA1*01:03/DPB1*04:01	EQEALEYFMKQMNDAHR	17	NetMHCIIpan	28.96	537.46	17.41
	HLA-DPA1*01:03/DPB1*04:01	EQEALEYFMKQMNDAHRG	18	NetMHCIIpan	59.19	581.18	17.41
	HLA-DRB1*04:01	EYFMKQMNDAHR	12	Consensus	8.9	-	6.37
	HLA-DRB1*04:01	EYFMKQMNDAHRG	13	Consensus	6.08	-	6.37
	HLA-DRB1*04:01	EYFMKQMNDAHRGG	14	Consensus	4.85	-	6.37
	HLA-DRB1*04:01	EYFMKQMNDAHRGGW	15	Consensus	5.2	-	6.37
	HLA-DRB3*02:02	EYFMKQMNDAHRGGW	15	NetMHCIIpan	15	929.56	16.40
	HLA-DRB1*04:01	EYFMKQMNDAHRGGWT	16	Consensus	6.93	-	6.37
	HLA-DRB3*01:01	HRGGWTTKMDWIFHT	15	Consensus	9.1	-	18.00
	HLA-DRB1*04:01	LEYFMKQMNDAHR	13	Consensus	6.08	-	6.37
	HLA-DRB1*04:01	LEYFMKQMNDAHRG	14	Consensus	4.85	-	6.37
	HLA-DRB3*02:02	LEYFMKQMNDAHRG	14	NetMHCIIpan	12.92	960.74	16.40
	HLA-DRB1*04:01	LEYFMKQMNDAHRGG	15	Consensus	5.2	-	6.37
	HLA-DRB3*02:02	LEYFMKQMNDAHRGG	15	NetMHCIIpan	14	842.99	16.40
	HLA-DRB1*04:01	LEYFMKQMNDAHRGGW	16	Consensus	6.93	-	6.37
	HLA-DRB3*02:02	LEYFMKQMNDAHRGGW	16	NetMHCIIpan	16.16	864.52	16.40
	HLA-DPA1*01:03/DPB1*04:01	QEALEYFMKQMNDAHR	16	NetMHCIIpan	21.93	703.46	17.41
	HLA-DRB1*04:01	QEALEYFMKQMNDAHR	16	Consensus	6.93	-	6.37
	HLA-DPA1*01:03/DPB1*04:01	QEALEYFMKQMNDAHRG	17	NetMHCIIpan	39.17	790.95	17.41
	HLA-DPA1*01:03/DPB1*04:01	QEALEYFMKQMNDAHRGG	18	NetMHCIIpan	79.91	831.41	17.41
	HLA-DRB3*01:01	RGGWTTKMDWIFHT	14	Consensus	7.97	-	18.00
	HLA-DRB3*01:01	RGGWTTKMDWIFHTI	15	Consensus	7.6	-	18.00
	HLA-DPA1*01:03/DPB1*04:01	RGGWTTKMDWIFHTIKQH	18	NetMHCIIpan	85.83	902.16	17.41
	HLA-DPA1*01:03/DPB1*04:01	TEQEALEYFMKQMNDAHR	18	NetMHCIIpan	47.35	466.35	17.41
	HLA-DRB1*04:01	YFMKQMNDAHRG	12	Consensus	9.79	-	6.37
	HLA-DRB1*04:01	YFMKQMNDAHRGG	13	Consensus	6.08	-	6.37
	HLA-DRB1*04:01	YFMKQMNDAHRGGW	14	Consensus	4.85	-	6.37
	HLA-DRB1*04:01	YFMKQMNDAHRGGWT	15	Consensus	5.8	-	6.37
	HLA-DRB1*04:01	YFMKQMNDAHRGGWTT	16	Consensus	8.43	-	6.37
PIK3CA N345K	HLA-DRB1*01:01	ALRIKILCATYV	12	Consensus	2.43	-	7.54
	HLA-DRB1*08:02	ALRIKILCATYV	12	Consensus	5.63	-	0.68
	HLA-DRB1*01:01	ALRIKILCATYVK	13	Consensus	2.03	-	7.54
	HLA-DRB1*07:01	ALRIKILCATYVK	13	Consensus	5.61	-	12.21
	HLA-DRB1*08:02	ALRIKILCATYVK	13	Consensus	4.52	-	0.68
	HLA-DRB1*12:01	ALRIKILCATYVK	13	Consensus	1.72	-	1.97
	HLA-DRB1*13:02	ALRIKILCATYVK	13	Consensus	8.73	-	4.60
	HLA-DRB1*15:01	ALRIKILCATYVK	13	Consensus	2.49	-	11.16
	HLA-DRB3*02:02	ALRIKILCATYVK	13	NetMHCIIpan	11.23	985.45	16.40
	HLA-DRB4*01:01	ALRIKILCATYVK	13	Consensus	6.39	-	28.00
	HLA-DRB5*01:01	ALRIKILCATYVK	13	Consensus	7.48	-	16.10
	HLA-DPA1*01:03/DPB1*02:01	ALRIKILCATYVKV	14	Consensus	7.32	-	15.31
	HLA-DPA1*01:03/DPB1*04:01	ALRIKILCATYVKV	14	NetMHCIIpan	3.77	281.81	17.41
	HLA-DRB1*01:01	ALRIKILCATYVKV	14	Consensus	1.72	-	7.54
	HLA-DRB1*04:05	ALRIKILCATYVKV	14	Consensus	9.58	-	1.16
	HLA-DRB1*07:01	ALRIKILCATYVKV	14	Consensus	4.2	-	12.21
	HLA-DRB1*08:02	ALRIKILCATYVKV	14	Consensus	4.2	-	0.68
	HLA-DRB1*12:01	ALRIKILCATYVKV	14	Consensus	1.56	-	1.97
	HLA-DRB1*13:02	ALRIKILCATYVKV	14	Consensus	5.38	-	4.60
	HLA-DRB1*15:01	ALRIKILCATYVKV	14	Consensus	1.94	-	11.16
	HLA-DRB3*02:02	ALRIKILCATYVKV	14	NetMHCIIpan	7.32	549.59	16.40
	HLA-DRB4*01:01	ALRIKILCATYVKV	14	Consensus	5.49	-	28.00
	HLA-DRB5*01:01	ALRIKILCATYVKV	14	Consensus	6.25	-	16.10
	HLA-DPA1*01:03/DPB1*02:01	ALRIKILCATYVKVN	15	Consensus	8.2	-	15.31
	HLA-DPA1*01:03/DPB1*04:01	ALRIKILCATYVKVN	15	NetMHCIIpan	4.5	229.91	17.41
	HLA-DPA1*02:01/DPB1*14:01	ALRIKILCATYVKVN	15	NetMHCIIpan	4.2	923.04	11.59
	HLA-DRB1*01:01	ALRIKILCATYVKVN	15	Consensus	2	-	7.54
	HLA-DRB1*04:05	ALRIKILCATYVKVN	15	Consensus	8.9	-	1.16
	HLA-DRB1*07:01	ALRIKILCATYVKVN	15	Consensus	5.4	-	12.21
	HLA-DRB1*08:02	ALRIKILCATYVKVN	15	Consensus	5.1	-	0.68
	HLA-DRB1*12:01	ALRIKILCATYVKVN	15	Consensus	1.9	-	1.97
	HLA-DRB1*13:02	ALRIKILCATYVKVN	15	Consensus	5.7	-	4.60
	HLA-DRB1*15:01	ALRIKILCATYVKVN	15	Consensus	2.7	-	11.16
	HLA-DRB3*02:02	ALRIKILCATYVKVN	15	NetMHCIIpan	9.1	560.67	16.40
	HLA-DRB4*01:01	ALRIKILCATYVKVN	15	Consensus	6.4	-	28.00
	HLA-DRB5*01:01	ALRIKILCATYVKVN	15	Consensus	7.1	-	16.10
	HLA-DPA1*01:03/DPB1*04:01	ALRIKILCATYVKVNI	16	NetMHCIIpan	5.08	192.35	17.41
	HLA-DPA1*02:01/DPB1*14:01	ALRIKILCATYVKVNI	16	NetMHCIIpan	6.12	759.96	11.59
	HLA-DRB1*01:01	ALRIKILCATYVKVNI	16	Consensus	3.46	-	7.54
	HLA-DRB1*07:01	ALRIKILCATYVKVNI	16	Consensus	7.96	-	12.21
	HLA-DRB1*08:02	ALRIKILCATYVKVNI	16	Consensus	7.62	-	0.68

HLA-DRB1*12:01	ALRIKILCATYVKVNI	16	Consensus	2.89	-	1.97
HLA-DRB1*13:02	ALRIKILCATYVKVNI	16	Consensus	7.5	-	4.60
HLA-DRB1*15:01	ALRIKILCATYVKVNI	16	Consensus	4.73	-	11.16
HLA-DRB3*02:02	ALRIKILCATYVKVNI	16	NetMHCIIpan	13.85	723.59	16.40
HLA-DRB4*01:01	ALRIKILCATYVKVNI	16	Consensus	8.43	-	28.00
HLA-DPA1*01:03/DPB1*04:01	ALRIKILCATYVKVNIR	17	NetMHCIIpan	9.54	212.59	17.41
HLA-DPA1*02:01/DPB1*14:01	ALRIKILCATYVKVNIR	17	NetMHCIIpan	11.07	715.8	11.59
HLA-DRB1*01:01	ALRIKILCATYVKVNIR	17	Consensus	5.96	-	7.54
HLA-DRB1*12:01	ALRIKILCATYVKVNIR	17	Consensus	5.71	-	1.97
HLA-DRB1*15:01	ALRIKILCATYVKVNIR	17	Consensus	9.71	-	11.16
HLA-DRB3*02:02	ALRIKILCATYVKVNIR	17	NetMHCIIpan	22.14	883.35	16.40
HLA-DPA1*01:03/DPB1*04:01	ALRIKILCATYVKVNIRD	18	NetMHCIIpan	21.01	236.3	17.41
HLA-DPA1*02:01/DPB1*14:01	ALRIKILCATYVKVNIRD	18	NetMHCIIpan	25.16	760.5	11.59
HLA-DRB1*04:01	ATPYMNGETSTKS	13	Consensus	5.61	-	6.37
HLA-DRB1*04:01	ATPYMNGETSTKSL	14	Consensus	4.52	-	6.37
HLA-DRB1*04:01	ATPYMNGETSTKSLW	15	Consensus	4.7	-	6.37
HLA-DRB1*04:01	ATPYMNGETSTKSLWV	16	Consensus	6.35	-	6.37
HLA-DRB1*01:01	ETSTKSLWVINSALRI	16	Consensus	6.81	-	7.54
HLA-DRB1*08:02	ETSTKSLWVINSALRI	16	Consensus	3.58	-	0.68
HLA-DRB1*15:01	ETSTKSLWVINSALRI	16	Consensus	5.43	-	11.16
HLA-DRB5*01:01	ETSTKSLWVINSALRI	16	Consensus	6.81	-	16.10
HLA-DRB1*08:02	ETSTKSLWVINSALRIK	17	Consensus	5.96	-	0.68
HLA-DRB1*12:01	ETSTKSLWVINSALRIK	17	Consensus	7.83	-	1.97
HLA-DRB1*15:01	ETSTKSLWVINSALRIK	17	Consensus	9.03	-	11.16
HLA-DRB1*12:01	ETSTKSLWVINSALRIKI	18	Consensus	9.62	-	1.97
HLA-DRB3*01:01	ETSTKSLWVINSALRIKI	18	Consensus	9.77	-	18.00
HLA-DRB1*08:02	FTMPSYSRRISTATP	15	Consensus	8.9	-	0.68
HLA-DRB1*08:02	GETSTKSLWVINSALRI	17	Consensus	5.96	-	0.68
HLA-DRB1*15:01	GETSTKSLWVINSALRI	17	Consensus	9.2	-	11.16
HLA-DPA1*01:03/DPB1*04:01	IKILCATYVKVN	12	NetMHCIIpan	8.9	751	17.41
HLA-DPA1*01:03/DPB1*02:01	IKILCATYVKVNI	13	Consensus	9.67	-	15.31
HLA-DPA1*01:03/DPB1*04:01	IKILCATYVKVNI	13	NetMHCIIpan	5.3	451.81	17.41
HLA-DRB1*01:01	IKILCATYVKVNI	13	Consensus	8.73	-	7.54
HLA-DRB1*07:01	IKILCATYVKVNI	13	Consensus	8.11	-	12.21
HLA-DRB1*15:01	IKILCATYVKVNI	13	Consensus	8.11	-	11.16
HLA-DPA1*01:03/DPB1*02:01	IKILCATYVKVNIR	14	Consensus	8.29	-	15.31
HLA-DPA1*01:03/DPB1*04:01	IKILCATYVKVNIR	14	NetMHCIIpan	4.74	340.4	17.41
HLA-DRB1*01:01	IKILCATYVKVNIR	14	Consensus	7.97	-	7.54
HLA-DRB1*07:01	IKILCATYVKVNIR	14	Consensus	7.65	-	12.21
HLA-DRB1*15:01	IKILCATYVKVNIR	14	Consensus	8.51	-	11.16
HLA-DPA1*01:03/DPB1*02:01	IKILCATYVKVNIRD	15	Consensus	9.4	-	15.31
HLA-DPA1*01:03/DPB1*04:01	IKILCATYVKVNIRD	15	NetMHCIIpan	6.5	307.43	17.41
HLA-DRB1*01:01	IKILCATYVKVNIRD	15	Consensus	9.3	-	7.54
HLA-DRB1*07:01	IKILCATYVKVNIRD	15	Consensus	9.7	-	12.21
HLA-DPA1*01:03/DPB1*02:01	IKILCATYVKVNIRDI	16	Consensus	9.93	-	15.31
HLA-DPA1*01:03/DPB1*04:01	IKILCATYVKVNIRDI	16	NetMHCIIpan	9.7	314.87	17.41
HLA-DRB1*01:01	IKILCATYVKVNIRDI	16	Consensus	4.39	-	7.54
HLA-DRB1*07:01	IKILCATYVKVNIRDI	16	Consensus	4.27	-	12.21
HLA-DRB1*09:01	IKILCATYVKVNIRDI	16	Consensus	0.91	-	1.68
HLA-DRB1*11:01	IKILCATYVKVNIRDI	16	Consensus	7.85	-	4.25
HLA-DRB1*13:02	IKILCATYVKVNIRDI	16	Consensus	4.85	-	4.60
HLA-DPA1*01:03/DPB1*04:01	IKILCATYVKVNIRDID	17	NetMHCIIpan	20.44	373.3	17.41
HLA-DRB1*01:01	IKILCATYVKVNIRDID	17	Consensus	7.49	-	7.54
HLA-DRB1*07:01	IKILCATYVKVNIRDID	17	Consensus	7.15	-	12.21
HLA-DRB1*09:01	IKILCATYVKVNIRDID	17	Consensus	1.62	-	1.68
HLA-DRB1*13:02	IKILCATYVKVNIRDID	17	Consensus	7.83	-	4.60
HLA-DPA1*01:03/DPB1*04:01	IKILCATYVKVNIRDIDK	18	NetMHCIIpan	41.44	407.26	17.41
HLA-DRB1*09:01	IKILCATYVKVNIRDIDK	18	Consensus	3.26	-	1.68
HLA-DRB1*13:02	ILCATYVKVNIRDIDK	16	Consensus	8.31	-	4.60
HLA-DRB1*01:01	INSALRIKILCATYV	15	Consensus	2.2	-	7.54
HLA-DRB1*08:02	INSALRIKILCATYV	15	Consensus	4	-	0.68
HLA-DRB1*13:02	INSALRIKILCATYV	15	Consensus	8.2	-	4.60
HLA-DPA1*01:03/DPB1*04:01	INSALRIKILCATYVK	16	NetMHCIIpan	21.93	708.94	17.41
HLA-DQA1*01:02/DQB1*06:02	INSALRIKILCATYVK	16	Consensus	3.12	-	10.40
HLA-DRB1*01:01	INSALRIKILCATYVK	16	Consensus	3.69	-	7.54
HLA-DRB1*07:01	INSALRIKILCATYVK	16	Consensus	8.43	-	12.21
HLA-DRB1*08:02	INSALRIKILCATYVK	16	Consensus	6.58	-	0.68
HLA-DRB1*12:01	INSALRIKILCATYVK	16	Consensus	3.12	-	1.97
HLA-DRB1*15:01	INSALRIKILCATYVK	16	Consensus	5.66	-	11.16
HLA-DRB4*01:01	INSALRIKILCATYVK	16	Consensus	6.58	-	28.00
HLA-DPA1*01:03/DPB1*04:01	INSALRIKILCATYVKV	17	NetMHCIIpan	11.41	243.57	17.41
HLA-DPA1*02:01/DPB1*14:01	INSALRIKILCATYVKV	17	NetMHCIIpan	13.29	808.99	11.59
HLA-DQA1*01:02/DQB1*06:02	INSALRIKILCATYVKV	17	Consensus	6.81	-	10.40
HLA-DRB1*01:01	INSALRIKILCATYVKV	17	Consensus	5.62	-	7.54
HLA-DRB1*12:01	INSALRIKILCATYVKV	17	Consensus	5.19	-	1.97
HLA-DRB1*15:01	INSALRIKILCATYVKV	17	Consensus	8.52	-	11.16
HLA-DPA1*01:03/DPB1*04:01	INSALRIKILCATYVKVN	18	NetMHCIIpan	23.97	257.86	17.41
HLA-DPA1*02:01/DPB1*14:01	INSALRIKILCATYVKVN	18	NetMHCIIpan	26.05	779.32	11.59
HLA-DRB1*12:01	INSALRIKILCATYVKVN	18	Consensus	9.17	-	1.97
HLA-DRB1*04:01	ISTATPYMNGETSTKS	16	Consensus	6.35	-	6.37
HLA-DPA1*01:03/DPB1*04:01	KILCATYVKVNI	12	NetMHCIIpan	10.38	850.18	17.41
HLA-DPA1*01:03/DPB1*04:01	KILCATYVKVNIR	13	NetMHCIIpan	7.64	641.18	17.41
HLA-DPA1*01:03/DPB1*04:01	KILCATYVKVNIRD	14	NetMHCIIpan	7.65	536.93	17.41
HLA-DPA1*01:03/DPB1*04:01	KILCATYVKVNIRDI	15	NetMHCIIpan	8.8	419.01	17.41
HLA-DPA1*01:03/DPB1*04:01	KILCATYVKVNIRDID	16	NetMHCIIpan	15.01	450.34	17.41
HLA-DRB1*13:02	KILCATYVKVNIRDID	16	Consensus	8.31	-	4.60
HLA-DPA1*01:03/DPB1*04:01	KILCATYVKVNIRDIDK	17	NetMHCIIpan	27.25	510.13	17.41
HLA-DPA1*01:03/DPB1*04:01	KILCATYVKVNIRDIDKI	18	NetMHCIIpan	56.23	528.01	17.41
HLA-DRB1*01:01	KSLWVINSALRI	12	Consensus	8.9	-	7.54
HLA-DRB1*08:02	KSLWVINSALRI	12	Consensus	5.34	-	0.68
HLA-DRB1*03:01	KSLWVINSALRIKI	14	Consensus	9.15	-	9.82
HLA-DRB1*11:01	KSLWVINSALRIKI	14	Consensus	3.98	-	4.25
HLA-DRB1*11:01	KSLWVINSALRIKIL	15	Consensus	4.1	-	4.25

HLA-DRB1*08:02	KSLWVINSALRIKILC	16	Consensus	3.58	-	0.68
HLA-DRB1*11:01	KSLWVINSALRIKILC	16	Consensus	7.16	-	4.25
HLA-DRB1*08:02	KSLWVINSALRIKILCA	17	Consensus	5.96	-	0.68
HLA-DRB1*15:01	KSLWVINSALRIKILCA	17	Consensus	8.01	-	11.16
HLA-DRB1*08:02	KSLWVINSALRIKILCAT	18	Consensus	5.62	-	0.68
HLA-DRB3*01:01	KSLWVINSALRIKILCAT	18	Consensus	9.47	-	18.00
HLA-DRB1*03:01	KVNIRDIDKIYVR	13	Consensus	9.82	-	9.82
HLA-DRB1*03:01	KVNIRDIDKIYVRT	14	Consensus	8.61	-	9.82
HLA-DRB1*03:01	KVNIRDIDKIYVRTG	15	Consensus	9.9	-	9.82
HLA-DRB1*11:01	KVNIRDIDKIYVRTG	15	Consensus	8.7	-	4.25
HLA-DRB1*01:01	LRIKILCATYVK	12	Consensus	2.58	-	7.54
HLA-DRB1*07:01	LRIKILCATYVK	12	Consensus	8.3	-	12.21
HLA-DRB1*08:02	LRIKILCATYVK	12	Consensus	8.01	-	0.68
HLA-DRB1*12:01	LRIKILCATYVK	12	Consensus	2.39	-	1.97
HLA-DRB1*15:01	LRIKILCATYVK	12	Consensus	3.26	-	11.16
HLA-DPA1*01:03/DPB1*02:01	LRIKILCATYVKV	13	Consensus	8.42	-	15.31
HLA-DPA1*01:03/DPB1*04:01	LRIKILCATYVKV	13	NetMHCIIpan	4.21	377.53	17.41
HLA-DRB1*01:01	LRIKILCATYVKV	13	Consensus	2.03	-	7.54
HLA-DRB1*07:01	LRIKILCATYVKV	13	Consensus	4.68	-	12.21
HLA-DRB1*08:02	LRIKILCATYVKV	13	Consensus	6.24	-	0.68
HLA-DRB1*12:01	LRIKILCATYVKV	13	Consensus	2.11	-	1.97
HLA-DRB1*13:02	LRIKILCATYVKV	13	Consensus	6.71	-	4.60
HLA-DRB1*15:01	LRIKILCATYVKV	13	Consensus	2.03	-	11.16
HLA-DRB3*02:02	LRIKILCATYVKV	13	NetMHCIIpan	7.8	679.95	16.40
HLA-DRB4*01:01	LRIKILCATYVKV	13	Consensus	7.48	-	28.00
HLA-DRB5*01:01	LRIKILCATYVKV	13	Consensus	7.33	-	16.10
HLA-DPA1*01:03/DPB1*02:01	LRIKILCATYVKVN	14	Consensus	7.11	-	15.31
HLA-DPA1*01:03/DPB1*04:01	LRIKILCATYVKVN	14	NetMHCIIpan	3.77	286.69	17.41
HLA-DRB1*01:01	LRIKILCATYVKVN	14	Consensus	1.94	-	7.54
HLA-DRB1*04:05	LRIKILCATYVKVN	14	Consensus	8.51	-	1.16
HLA-DRB1*07:01	LRIKILCATYVKVN	14	Consensus	4.63	-	12.21
HLA-DRB1*08:02	LRIKILCATYVKVN	14	Consensus	5.92	-	0.68
HLA-DRB1*12:01	LRIKILCATYVKVN	14	Consensus	1.88	-	1.97
HLA-DRB1*13:02	LRIKILCATYVKVN	14	Consensus	5.38	-	4.60
HLA-DRB1*15:01	LRIKILCATYVKVN	14	Consensus	2.15	-	11.16
HLA-DRB3*02:02	LRIKILCATYVKVN	14	NetMHCIIpan	7	533.12	16.40
HLA-DRB4*01:01	LRIKILCATYVKVN	14	Consensus	7.43	-	28.00
HLA-DRB5*01:01	LRIKILCATYVKVN	14	Consensus	6.35	-	16.10
HLA-DPA1*01:03/DPB1*02:01	LRIKILCATYVKVNI	15	Consensus	8	-	15.31
HLA-DPA1*01:03/DPB1*04:01	LRIKILCATYVKVNI	15	NetMHCIIpan	3.5	190.24	17.41
HLA-DRB1*01:01	LRIKILCATYVKVNI	15	Consensus	2.2	-	7.54
HLA-DRB1*07:01	LRIKILCATYVKVNI	15	Consensus	5.6	-	12.21
HLA-DRB1*08:02	LRIKILCATYVKVNI	15	Consensus	7.1	-	0.68
HLA-DRB1*12:01	LRIKILCATYVKVNI	15	Consensus	2.4	-	1.97
HLA-DRB1*13:02	LRIKILCATYVKVNI	15	Consensus	5.7	-	4.60
HLA-DRB1*15:01	LRIKILCATYVKVNI	15	Consensus	3.1	-	11.16
HLA-DRB3*02:02	LRIKILCATYVKVNI	15	NetMHCIIpan	9.4	579.2	16.40
HLA-DRB4*01:01	LRIKILCATYVKVNI	15	Consensus	8.2	-	28.00
HLA-DRB5*01:01	LRIKILCATYVKVNI	15	Consensus	7.3	-	16.10
HLA-DPA1*01:03/DPB1*04:01	LRIKILCATYVKVNIR	16	NetMHCIIpan	5.19	195.64	17.41
HLA-DPA1*02:01/DPB1*14:01	LRIKILCATYVKVNIR	16	NetMHCIIpan	7.27	830.02	11.59
HLA-DRB1*01:01	LRIKILCATYVKVNIR	16	Consensus	3.69	-	7.54
HLA-DRB1*07:01	LRIKILCATYVKVNIR	16	Consensus	8.89	-	12.21
HLA-DRB1*08:02	LRIKILCATYVKVNIR	16	Consensus	8.89	-	0.68
HLA-DRB1*12:01	LRIKILCATYVKVNIR	16	Consensus	3.81	-	1.97
HLA-DRB1*13:02	LRIKILCATYVKVNIR	16	Consensus	7.5	-	4.60
HLA-DRB1*15:01	LRIKILCATYVKVNIR	16	Consensus	5.66	-	11.16
HLA-DRB3*02:02	LRIKILCATYVKVNIR	16	NetMHCIIpan	13.85	758.64	16.40
HLA-DPA1*01:03/DPB1*04:01	LRIKILCATYVKVNIRD	17	NetMHCIIpan	10.39	228.72	17.41
HLA-DPA1*02:01/DPB1*14:01	LRIKILCATYVKVNIRD	17	NetMHCIIpan	16.01	905.64	11.59
HLA-DRB1*01:01	LRIKILCATYVKVNIRD	17	Consensus	6.3	-	7.54
HLA-DRB1*12:01	LRIKILCATYVKVNIRD	17	Consensus	7.15	-	1.97
HLA-DPA1*01:03/DPB1*04:01	LRIKILCATYVKVNIRDI	18	NetMHCIIpan	21.01	234.8	17.41
HLA-DPA1*02:01/DPB1*14:01	LRIKILCATYVKVNIRDI	18	NetMHCIIpan	32.56	884.3	11.59
HLA-DRB1*09:01	LRIKILCATYVKVNIRDI	18	Consensus	2.9	-	1.68
HLA-DRB1*08:02	LWVINSALRIKI	12	Consensus	5.34	-	0.68
HLA-DRB1*11:01	LWVINSALRIKI	12	Consensus	6.82	-	4.25
HLA-DRB1*08:02	LWVINSALRIKIL	13	Consensus	4.05	-	0.68
HLA-DRB1*11:01	LWVINSALRIKIL	13	Consensus	4.83	-	4.25
HLA-DRB1*03:01	LWVINSALRIKILC	14	Consensus	9.15	-	9.82
HLA-DRB1*08:02	LWVINSALRIKILC	14	Consensus	3.98	-	0.68
HLA-DRB1*11:01	LWVINSALRIKILC	14	Consensus	4.41	-	4.25
HLA-DRB1*15:01	LWVINSALRIKILC	14	Consensus	8.94	-	11.16
HLA-DRB1*08:02	LWVINSALRIKILCA	15	Consensus	4.2	-	0.68
HLA-DRB1*11:01	LWVINSALRIKILCA	15	Consensus	4.9	-	4.25
HLA-DRB1*15:01	LWVINSALRIKILCA	15	Consensus	9.6	-	11.16
HLA-DRB1*08:02	LWVINSALRIKILCAT	16	Consensus	1.96	-	0.68
HLA-DRB1*11:01	LWVINSALRIKILCAT	16	Consensus	7.04	-	4.25
HLA-DRB1*15:01	LWVINSALRIKILCAT	16	Consensus	4.85	-	11.16
HLA-DRB3*01:01	LWVINSALRIKILCAT	16	Consensus	3.23	-	18.00
HLA-DRB5*01:01	LWVINSALRIKILCAT	16	Consensus	6.81	-	16.10
HLA-DRB1*08:02	LWVINSALRIKILCATY	17	Consensus	2.9	-	0.68
HLA-DRB1*15:01	LWVINSALRIKILCATY	17	Consensus	8.35	-	11.16
HLA-DRB3*01:01	LWVINSALRIKILCATY	17	Consensus	5.45	-	18.00
HLA-DRB1*01:01	LWVINSALRIKILCATYV	18	Consensus	6.22	-	7.54
HLA-DRB1*08:02	LWVINSALRIKILCATYV	18	Consensus	3.85	-	0.68
HLA-DRB1*15:01	LWVINSALRIKILCATYV	18	Consensus	9.77	-	11.16
HLA-DRB1*08:02	MPSYSRRISTATPY	14	Consensus	9.05	-	0.68
HLA-DRB1*11:01	NIRDIDKIYVRTGI	14	Consensus	8.4	-	4.25
HLA-DRB1*01:01	NSALRIKILCATYV	14	Consensus	1.94	-	7.54
HLA-DRB1*08:02	NSALRIKILCATYV	14	Consensus	3.55	-	0.68
HLA-DRB1*13:02	NSALRIKILCATYV	14	Consensus	7.75	-	4.60
HLA-DPA1*01:03/DPB1*04:01	NSALRIKILCATYVK	15	NetMHCIIpan	17	796.51	17.41

HLA-DQA1*01:02/DQB1*06:02	NSALRIKILCATYVK	15	Consensus	5.1	-	10.40
HLA-DRB1*01:01	NSALRIKILCATYVK	15	Consensus	2	-	7.54
HLA-DRB1*04:05	NSALRIKILCATYVK	15	Consensus	8.9	-	1.16
HLA-DRB1*07:01	NSALRIKILCATYVK	15	Consensus	6	-	12.21
HLA-DRB1*08:02	NSALRIKILCATYVK	15	Consensus	4.1	-	0.68
HLA-DRB1*12:01	NSALRIKILCATYVK	15	Consensus	2.05	-	1.97
HLA-DRB1*13:02	NSALRIKILCATYVK	15	Consensus	8.7	-	4.60
HLA-DRB1*15:01	NSALRIKILCATYVK	15	Consensus	3.8	-	11.16
HLA-DRB4*01:01	NSALRIKILCATYVK	15	Consensus	4.6	-	28.00
HLA-DRB5*01:01	NSALRIKILCATYVK	15	Consensus	7.6	-	16.10
HLA-DPA1*01:03/DPB1*04:01	NSALRIKILCATYVKV	16	NetMHCIIpan	6.46	227.42	17.41
HLA-DPA1*02:01/DPB1*14:01	NSALRIKILCATYVKV	16	NetMHCIIpan	6.93	812.64	11.59
HLA-DQA1*01:02/DQB1*06:02	NSALRIKILCATYVKV	16	Consensus	9.12	-	10.40
HLA-DRB1*01:01	NSALRIKILCATYVKV	16	Consensus	3.46	-	7.54
HLA-DRB1*07:01	NSALRIKILCATYVKV	16	Consensus	7.04	-	12.21
HLA-DRB1*08:02	NSALRIKILCATYVKV	16	Consensus	7.73	-	0.68
HLA-DRB1*12:01	NSALRIKILCATYVKV	16	Consensus	3.23	-	1.97
HLA-DRB1*13:02	NSALRIKILCATYVKV	16	Consensus	7.5	-	4.60
HLA-DRB1*15:01	NSALRIKILCATYVKV	16	Consensus	4.73	-	11.16
HLA-DRB3*02:02	NSALRIKILCATYVKV	16	NetMHCIIpan	17.31	927.44	16.40
HLA-DRB4*01:01	NSALRIKILCATYVKV	16	Consensus	6.81	-	28.00
HLA-DPA1*01:03/DPB1*04:01	NSALRIKILCATYVKVN	17	NetMHCIIpan	11.75	249.33	17.41
HLA-DPA1*02:01/DPB1*14:01	NSALRIKILCATYVKVN	17	NetMHCIIpan	12.77	788.03	11.59
HLA-DRB1*01:01	NSALRIKILCATYVKVN	17	Consensus	5.96	-	7.54
HLA-DRB1*12:01	NSALRIKILCATYVKVN	17	Consensus	5.19	-	1.97
HLA-DRB1*15:01	NSALRIKILCATYVKVN	17	Consensus	8.86	-	11.16
HLA-DPA1*01:03/DPB1*04:01	NSALRIKILCATYVKVNI	18	NetMHCIIpan	19.53	224.99	17.41
HLA-DPA1*02:01/DPB1*14:01	NSALRIKILCATYVKVNI	18	NetMHCIIpan	24.86	756.1	11.59
HLA-DRB1*04:01	PYMNGETSTKSL	12	Consensus	9.49	-	6.37
HLA-DRB1*04:01	PYMNGETSTKSLW	13	Consensus	8.11	-	6.37
HLA-DRB1*04:01	PYMNGETSTKSLWV	14	Consensus	7.11	-	6.37
HLA-DRB1*04:01	PYMNGETSTKSLWVI	15	Consensus	8.6	-	6.37
HLA-DRB1*04:01	PYMNGETSTKSLWVIN	16	Consensus	6.46	-	6.37
HLA-DPA1*01:03/DPB1*04:01	RIKILCATYVKV	12	NetMHCIIpan	5.63	514.7	17.41
HLA-DRB1*01:01	RIKILCATYVKV	12	Consensus	5.04	-	7.54
HLA-DRB1*12:01	RIKILCATYVKV	12	Consensus	6.82	-	1.97
HLA-DRB1*15:01	RIKILCATYVKV	12	Consensus	3.56	-	11.16
HLA-DRB3*02:02	RIKILCATYVKV	12	NetMHCIIpan	10.68	976.07	16.40
HLA-DPA1*01:03/DPB1*02:01	RIKILCATYVKVN	13	Consensus	8.89	-	15.31
HLA-DPA1*01:03/DPB1*04:01	RIKILCATYVKVN	13	NetMHCIIpan	4.68	407.54	17.41
HLA-DRB1*01:01	RIKILCATYVKVN	13	Consensus	4.21	-	7.54
HLA-DRB1*07:01	RIKILCATYVKVN	13	Consensus	7.95	-	12.21
HLA-DRB1*12:01	RIKILCATYVKVN	13	Consensus	4.83	-	1.97
HLA-DRB1*15:01	RIKILCATYVKVN	13	Consensus	2.81	-	11.16
HLA-DRB3*02:02	RIKILCATYVKVN	13	NetMHCIIpan	8.26	710.19	16.40
HLA-DPA1*01:03/DPB1*02:01	RIKILCATYVKVNI	14	Consensus	7.43	-	15.31
HLA-DPA1*01:03/DPB1*04:01	RIKILCATYVKVNI	14	NetMHCIIpan	3.23	248.03	17.41
HLA-DRB1*01:01	RIKILCATYVKVNI	14	Consensus	5.28	-	7.54
HLA-DRB1*07:01	RIKILCATYVKVNI	14	Consensus	7.11	-	12.21
HLA-DRB1*12:01	RIKILCATYVKVNI	14	Consensus	4.36	-	1.97
HLA-DRB1*15:01	RIKILCATYVKVNI	14	Consensus	2.91	-	11.16
HLA-DRB3*02:02	RIKILCATYVKVNI	14	NetMHCIIpan	7.86	593.17	16.40
HLA-DPA1*01:03/DPB1*02:01	RIKILCATYVKVNIR	15	Consensus	8.3	-	15.31
HLA-DPA1*01:03/DPB1*04:01	RIKILCATYVKVNIR	15	NetMHCIIpan	3.8	203.42	17.41
HLA-DRB1*01:01	RIKILCATYVKVNIR	15	Consensus	7.5	-	7.54
HLA-DRB1*07:01	RIKILCATYVKVNIR	15	Consensus	7.9	-	12.21
HLA-DRB1*12:01	RIKILCATYVKVNIR	15	Consensus	5.6	-	1.97
HLA-DRB1*15:01	RIKILCATYVKVNIR	15	Consensus	4.4	-	11.16
HLA-DRB3*02:02	RIKILCATYVKVNIR	15	NetMHCIIpan	11	650.26	16.40
HLA-DPA1*01:03/DPB1*04:01	RIKILCATYVKVNIRD	16	NetMHCIIpan	6.23	222.45	17.41
HLA-DPA1*02:01/DPB1*14:01	RIKILCATYVKVNIRD	16	NetMHCIIpan	9.47	979.06	11.59
HLA-DRB1*01:01	RIKILCATYVKVNIRD	16	Consensus	4.16	-	7.54
HLA-DRB1*07:01	RIKILCATYVKVNIRD	16	Consensus	9.7	-	12.21
HLA-DRB1*12:01	RIKILCATYVKVNIRD	16	Consensus	6.75	-	1.97
HLA-DRB1*15:01	RIKILCATYVKVNIRD	16	Consensus	7.96	-	11.16
HLA-DRB3*02:02	RIKILCATYVKVNIRD	16	NetMHCIIpan	17.31	962.33	16.40
HLA-DPA1*01:03/DPB1*04:01	RIKILCATYVKVNIRDI	17	NetMHCIIpan	11.24	240.54	17.41
HLA-DPA1*02:01/DPB1*14:01	RIKILCATYVKVNIRDI	17	NetMHCIIpan	18.74	986.48	11.59
HLA-DRB1*01:01	RIKILCATYVKVNIRDI	17	Consensus	7.15	-	7.54
HLA-DRB1*07:01	RIKILCATYVKVNIRDI	17	Consensus	6.98	-	12.21
HLA-DRB1*09:01	RIKILCATYVKVNIRDI	17	Consensus	1.53	-	1.68
HLA-DRB1*13:02	RIKILCATYVKVNIRDI	17	Consensus	7.83	-	4.60
HLA-DPA1*01:03/DPB1*04:01	RIKILCATYVKVNIRDID	18	NetMHCIIpan	25.16	268.14	17.41
HLA-DRB1*09:01	RIKILCATYVKVNIRDID	18	Consensus	3.26	-	1.68
HLA-DRB1*01:01	SALRIKILCATYV	13	Consensus	2.03	-	7.54
HLA-DRB1*08:02	SALRIKILCATYV	13	Consensus	4.05	-	0.68
HLA-DRB1*13:02	SALRIKILCATYV	13	Consensus	8.73	-	4.60
HLA-DPA1*01:03/DPB1*04:01	SALRIKILCATYVK	14	NetMHCIIpan	14	978.7	17.41
HLA-DRB1*01:01	SALRIKILCATYVK	14	Consensus	1.94	-	7.54
HLA-DRB1*04:05	SALRIKILCATYVK	14	Consensus	8.08	-	1.16
HLA-DRB1*07:01	SALRIKILCATYVK	14	Consensus	5.06	-	12.21
HLA-DRB1*08:02	SALRIKILCATYVK	14	Consensus	3.88	-	0.68
HLA-DRB1*12:01	SALRIKILCATYVK	14	Consensus	1.56	-	1.97
HLA-DRB1*13:02	SALRIKILCATYVK	14	Consensus	7.65	-	4.60
HLA-DRB1*15:01	SALRIKILCATYVK	14	Consensus	2.48	-	11.16
HLA-DRB3*02:02	SALRIKILCATYVK	14	NetMHCIIpan	11.84	877.8	16.40
HLA-DRB4*01:01	SALRIKILCATYVK	14	Consensus	5.71	-	28.00
HLA-DRB5*01:01	SALRIKILCATYVK	14	Consensus	6.46	-	16.10
HLA-DPA1*01:03/DPB1*02:01	SALRIKILCATYVKV	15	Consensus	8.6	-	15.31
HLA-DPA1*01:03/DPB1*04:01	SALRIKILCATYVKV	15	NetMHCIIpan	4.5	228.7	17.41
HLA-DPA1*02:01/DPB1*14:01	SALRIKILCATYVKV	15	NetMHCIIpan	4.4	932.51	11.59
HLA-DRB1*01:01	SALRIKILCATYVKV	15	Consensus	2	-	7.54
HLA-DRB1*04:05	SALRIKILCATYVKV	15	Consensus	8.9	-	1.16

HLA-DRB1*07:01	SALRIKILCATYVKV	15	Consensus	4.9	-	12.21
HLA-DRB1*08:02	SALRIKILCATYVKV	15	Consensus	4.4	-	0.68
HLA-DRB1*12:01	SALRIKILCATYVKV	15	Consensus	1.8	-	1.97
HLA-DRB1*13:02	SALRIKILCATYVKV	15	Consensus	5.7	-	4.60
HLA-DRB1*15:01	SALRIKILCATYVKV	15	Consensus	2.6	-	11.16
HLA-DRB3*02:02	SALRIKILCATYVKV	15	NetMHCIIpan	11	639.82	16.40
HLA-DRB4*01:01	SALRIKILCATYVKV	15	Consensus	6.2	-	28.00
HLA-DRB5*01:01	SALRIKILCATYVKV	15	Consensus	7.1	-	16.10
HLA-DPA1*01:03/DPB1*04:01	SALRIKILCATYVKVN	16	NetMHCIIpan	6.46	226.93	17.41
HLA-DPA1*02:01/DPB1*14:01	SALRIKILCATYVKVN	16	NetMHCIIpan	6.58	792.9	11.59
HLA-DRB1*01:01	SALRIKILCATYVKVN	16	Consensus	3.35	-	7.54
HLA-DRB1*07:01	SALRIKILCATYVKVN	16	Consensus	7.73	-	12.21
HLA-DRB1*08:02	SALRIKILCATYVKVN	16	Consensus	7.62	-	0.68
HLA-DRB1*12:01	SALRIKILCATYVKVN	16	Consensus	2.89	-	1.97
HLA-DRB1*13:02	SALRIKILCATYVKVN	16	Consensus	7.5	-	4.60
HLA-DRB1*15:01	SALRIKILCATYVKVN	16	Consensus	4.5	-	11.16
HLA-DRB3*02:02	SALRIKILCATYVKVN	16	NetMHCIIpan	15.01	785.89	16.40
HLA-DRB4*01:01	SALRIKILCATYVKVN	16	Consensus	7.5	-	28.00
HLA-DPA1*01:03/DPB1*04:01	SALRIKILCATYVKVNI	17	NetMHCIIpan	9.37	210.78	17.41
HLA-DPA1*02:01/DPB1*14:01	SALRIKILCATYVKVNI	17	NetMHCIIpan	12.26	763.95	11.59
HLA-DRB1*01:01	SALRIKILCATYVKVNI	17	Consensus	5.79	-	7.54
HLA-DRB1*12:01	SALRIKILCATYVKVNI	17	Consensus	5.11	-	1.97
HLA-DRB1*15:01	SALRIKILCATYVKVNI	17	Consensus	8.86	-	11.16
HLA-DRB3*02:02	SALRIKILCATYVKVNI	17	NetMHCIIpan	23.85	935.3	16.40
HLA-DPA1*01:03/DPB1*04:01	SALRIKILCATYVKVNIR	18	NetMHCIIpan	19.24	223.06	17.41
HLA-DPA1*02:01/DPB1*14:01	SALRIKILCATYVKVNIR	18	NetMHCIIpan	23.09	711.22	11.59
HLA-DRB3*02:02	SALRIKILCATYVKVNIR	18	NetMHCIIpan	44.4	978.08	16.40
HLA-DRB1*01:01	SLWVINSALRIK	12	Consensus	8.9	-	7.54
HLA-DRB1*08:02	SLWVINSALRIK	12	Consensus	7.12	-	0.68
HLA-DRB1*15:01	SLWVINSALRIK	12	Consensus	8.9	-	11.16
HLA-DRB1*11:01	SLWVINSALRIKI	13	Consensus	4.52	-	4.25
HLA-DRB1*03:01	SLWVINSALRIKIL	14	Consensus	9.15	-	9.82
HLA-DRB1*11:01	SLWVINSALRIKIL	14	Consensus	3.66	-	4.25
HLA-DRB1*08:02	SLWVINSALRIKILC	15	Consensus	4.2	-	0.68
HLA-DRB1*11:01	SLWVINSALRIKILC	15	Consensus	4.3	-	4.25
HLA-DRB1*15:01	SLWVINSALRIKILC	15	Consensus	5.8	-	11.16
HLA-DRB1*08:02	SLWVINSALRIKILCA	16	Consensus	3.58	-	0.68
HLA-DRB1*11:01	SLWVINSALRIKILCA	16	Consensus	6.93	-	4.25
HLA-DRB1*15:01	SLWVINSALRIKILCA	16	Consensus	4.85	-	11.16
HLA-DRB1*08:02	SLWVINSALRIKILCAT	17	Consensus	2.9	-	0.68
HLA-DRB1*15:01	SLWVINSALRIKILCAT	17	Consensus	8.18	-	11.16
HLA-DRB3*01:01	SLWVINSALRIKILCAT	17	Consensus	5.28	-	18.00
HLA-DRB1*08:02	SLWVINSALRIKILCATY	18	Consensus	5.33	-	0.68
HLA-DRB3*01:01	SLWVINSALRIKILCATY	18	Consensus	9.77	-	18.00
HLA-DRB1*04:01	STATPYMNGETSTKS	15	Consensus	4.7	-	6.37
HLA-DRB1*04:01	STATPYMNGETSTKSL	16	Consensus	6.35	-	6.37
HLA-DRB1*01:01	STKSLWVINSALRI	14	Consensus	4.52	-	7.54
HLA-DRB1*03:01	STKSLWVINSALRI	14	Consensus	9.15	-	9.82
HLA-DRB1*11:01	STKSLWVINSALRIKI	16	Consensus	7.5	-	4.25
HLA-DRB1*08:02	STKSLWVINSALRIKIL	17	Consensus	5.96	-	0.68
HLA-DRB1*15:01	STKSLWVINSALRIKIL	17	Consensus	7.83	-	11.16
HLA-DRB3*01:01	STKSLWVINSALRIKILC	18	Consensus	9.47	-	18.00
HLA-DRB1*04:01	TATPYMNGETSTKS	14	Consensus	4.52	-	6.37
HLA-DRB1*04:01	TATPYMNGETSTKSL	15	Consensus	4.7	-	6.37
HLA-DRB1*04:01	TATPYMNGETSTKSLW	16	Consensus	6.35	-	6.37
HLA-DRB1*01:01	TKSLWVINSALRI	13	Consensus	5.61	-	7.54
HLA-DRB1*03:01	TKSLWVINSALRIK	14	Consensus	9.15	-	9.82
HLA-DRB1*11:01	TKSLWVINSALRIKI	15	Consensus	4.5	-	4.25
HLA-DRB1*11:01	TKSLWVINSALRIKIL	16	Consensus	6.93	-	4.25
HLA-DRB1*08:02	TKSLWVINSALRIKILC	17	Consensus	5.96	-	0.68
HLA-DRB1*15:01	TKSLWVINSALRIKILC	17	Consensus	7.83	-	11.16
HLA-DRB3*01:01	TKSLWVINSALRIKILCA	18	Consensus	9.47	-	18.00
HLA-DRB1*08:02	TMPSYSRRISTATP	14	Consensus	8.94	-	0.68
HLA-DRB1*04:01	TPYMNGETSTKS	12	Consensus	8.9	-	6.37
HLA-DRB1*04:01	TPYMNGETSTKSL	13	Consensus	5.61	-	6.37
HLA-DRB1*04:01	TPYMNGETSTKSLW	14	Consensus	4.52	-	6.37
HLA-DRB1*04:01	TPYMNGETSTKSLWV	15	Consensus	4.7	-	6.37
HLA-DRB1*04:01	TPYMNGETSTKSLWVI	16	Consensus	6.35	-	6.37
HLA-DRB1*01:01	TSTKSLWVINSALRI	15	Consensus	4.8	-	7.54
HLA-DRB1*08:02	TSTKSLWVINSALRI	15	Consensus	2.7	-	0.68
HLA-DRB1*08:02	TSTKSLWVINSALRIK	16	Consensus	3.58	-	0.68
HLA-DRB1*15:01	TSTKSLWVINSALRIK	16	Consensus	5.19	-	11.16
HLA-DRB1*08:02	TSTKSLWVINSALRIKI	17	Consensus	5.96	-	0.68
HLA-DRB1*15:01	TSTKSLWVINSALRIKI	17	Consensus	8.18	-	11.16
HLA-DRB3*01:01	TSTKSLWVINSALRIKIL	18	Consensus	9.47	-	18.00
HLA-DRB1*01:01	VINSALRIKILCATYV	16	Consensus	3.69	-	7.54
HLA-DRB1*08:02	VINSALRIKILCATYV	16	Consensus	6.46	-	0.68
HLA-DPA1*01:03/DPB1*04:01	VINSALRIKILCATYVK	17	NetMHCIIpan	35.77	669.67	17.41
HLA-DQA1*01:02/DQB1*06:02	VINSALRIKILCATYVK	17	Consensus	4.26	-	10.40
HLA-DRB1*01:01	VINSALRIKILCATYVK	17	Consensus	6.13	-	7.54
HLA-DRB1*12:01	VINSALRIKILCATYVK	17	Consensus	4.09	-	1.97
HLA-DPA1*01:03/DPB1*04:01	VINSALRIKILCATYVKV	18	NetMHCIIpan	23.09	250.89	17.41
HLA-DPA1*02:01/DPB1*14:01	VINSALRIKILCATYVKV	18	NetMHCIIpan	24.86	756.7	11.59
HLA-DQA1*01:02/DQB1*06:02	VINSALRIKILCATYVKV	18	Consensus	9.77	-	10.40
HLA-DRB1*12:01	VINSALRIKILCATYVKV	18	Consensus	7.55	-	1.97
HLA-DRB1*03:01	VKVNIRDIDKIYVR	14	Consensus	8.18	-	9.82
HLA-DRB1*03:01	VKVNIRDIDKIYVRT	15	Consensus	9.3	-	9.82
HLA-DRB1*11:01	VNIRDIDKIYVRTG	14	Consensus	8.08	-	4.25
HLA-DRB1*11:01	VNIRDIDKIYVRTGI	15	Consensus	8.7	-	4.25
HLA-DRB1*11:01	WVINSALRIKIL	12	Consensus	6.82	-	4.25
HLA-DRB3*01:01	WVINSALRIKIL	12	Consensus	9.19	-	18.00
HLA-DRB1*11:01	WVINSALRIKILC	13	Consensus	5.46	-	4.25
HLA-DRB3*01:01	WVINSALRIKILC	13	Consensus	6.39	-	18.00

	HLA-DRB1*01:01	WVINSALRIKILCA	14	Consensus	9.91	-	7.54
	HLA-DRB1*03:01	WVINSALRIKILCA	14	Consensus	9.15	-	9.82
	HLA-DRB1*11:01	WVINSALRIKILCA	14	Consensus	4.52	-	4.25
	HLA-DRB3*01:01	WVINSALRIKILCA	14	Consensus	5.81	-	18.00
	HLA-DRB1*11:01	WVINSALRIKILCAT	15	Consensus	5.2	-	4.25
	HLA-DRB3*01:01	WVINSALRIKILCAT	15	Consensus	6.8	-	18.00
	HLA-DRB1*11:01	WVINSALRIKILCATY	16	Consensus	7.5	-	4.25
	HLA-DRB3*01:01	WVINSALRIKILCATY	16	Consensus	3.35	-	18.00
	HLA-DRB1*01:01	WVINSALRIKILCATYV	17	Consensus	3.41	-	7.54
	HLA-DRB1*11:01	WVINSALRIKILCATYV	17	Consensus	6.13	-	4.25
	HLA-DRB1*15:01	WVINSALRIKILCATYV	17	Consensus	5.11	-	11.16
	HLA-DRB3*01:01	WVINSALRIKILCATYV	17	Consensus	5.62	-	18.00
	HLA-DPA1*01:03/DPB1*04:01	WVINSALRIKILCATYVK	18	NetMHCIIpan	41.44	397.76	17.41
	HLA-DPA1*02:01/DPB1*14:01	WVINSALRIKILCATYVK	18	NetMHCIIpan	26.05	781.41	11.59
	HLA-DQA1*01:02/DQB1*06:02	WVINSALRIKILCATYVK	18	Consensus	7.7	-	10.40
	HLA-DRB1*01:01	WVINSALRIKILCATYVK	18	Consensus	6.22	-	7.54
	HLA-DRB1*12:01	WVINSALRIKILCATYVK	18	Consensus	3.66	-	1.97
	HLA-DRB1*13:02	WVINSALRIKILCATYVK	18	Consensus	2.46	-	4.60
	HLA-DRB1*15:01	WVINSALRIKILCATYVK	18	Consensus	9.77	-	11.16
	HLA-DRB3*02:02	WVINSALRIKILCATYVK	18	NetMHCIIpan	6.51	146.81	16.40
	HLA-DRB1*04:01	YMNGETSTKSLWVINS	16	Consensus	6.35	-	6.37
	HLA-DRB1*03:01	YVKVNIRDIDKIYVR	15	Consensus	9.4	-	9.82
TP53 R196*	-	-	-	-	-	-	-
TP53 R175H	HLA-DQA1*01:02/DQB1*06:02	KQSQHMTEVVRHCP	14	Consensus	8.2	-	10.40
	HLA-DQA1*01:02/DQB1*06:02	KQSQHMTEVVRHCPH	15	Consensus	8	-	10.40
	HLA-DQA1*01:02/DQB1*06:02	QSQHMTEVVRHCPH	14	Consensus	7.4	-	10.40
TP53 Y220C	HLA-DRB1*04:05	DDRNTFRHSVVVPC	14	Consensus	6.57	-	1.16
	HLA-DRB1*07:01	DDRNTFRHSVVVPC	14	Consensus	4.74	-	12.21
	HLA-DRB3*02:02	DDRNTFRHSVVVPC	14	NetMHCIIpan	7.32	549.98	16.40
	HLA-DRB1*04:05	DDRNTFRHSVVVPCE	15	Consensus	7.5	-	1.16
	HLA-DRB1*07:01	DDRNTFRHSVVVPCE	15	Consensus	5	-	12.21
	HLA-DRB3*02:02	DDRNTFRHSVVVPCE	15	NetMHCIIpan	5.7	342.08	16.40
	HLA-DRB1*07:01	DDRNTFRHSVVVPCEP	16	Consensus	6.46	-	12.21
	HLA-DRB3*02:02	DDRNTFRHSVVVPCEP	16	NetMHCIIpan	8.31	434.46	16.40
	HLA-DRB3*02:02	DDRNTFRHSVVVPCEPP	17	NetMHCIIpan	15.67	598.28	16.40
	HLA-DRB3*02:02	DDRNTFRHSVVVPCEPPE	18	NetMHCIIpan	32.56	677.58	16.40
	HLA-DRB1*04:05	DRNTFRHSVVVPC	13	Consensus	7.17	-	1.16
	HLA-DRB1*07:01	DRNTFRHSVVVPC	13	Consensus	5.77	-	12.21
	HLA-DRB3*02:02	DRNTFRHSVVVPC	13	NetMHCIIpan	7.02	601.87	16.40
	HLA-DRB1*04:05	DRNTFRHSVVVPCE	14	Consensus	6.35	-	1.16
	HLA-DRB1*07:01	DRNTFRHSVVVPCE	14	Consensus	4.74	-	12.21
	HLA-DRB3*02:02	DRNTFRHSVVVPCE	14	NetMHCIIpan	4.52	330.49	16.40
	HLA-DRB1*04:05	DRNTFRHSVVVPCEP	15	Consensus	8	-	1.16
	HLA-DRB1*07:01	DRNTFRHSVVVPCEP	15	Consensus	5	-	12.21
	HLA-DRB3*02:02	DRNTFRHSVVVPCEP	15	NetMHCIIpan	5.4	319.65	16.40
	HLA-DRB1*07:01	DRNTFRHSVVVPCEPP	16	Consensus	6.46	-	12.21
	HLA-DRB3*02:02	DRNTFRHSVVVPCEPP	16	NetMHCIIpan	8.43	438.47	16.40
	HLA-DRB3*02:02	DRNTFRHSVVVPCEPPE	17	NetMHCIIpan	15.5	591.96	16.40
	HLA-DRB3*02:02	DRNTFRHSVVVPCEPPEV	18	NetMHCIIpan	32.56	676.55	16.40
	HLA-DRB3*02:02	EYLDDRNTFRHSVVVPC	17	NetMHCIIpan	22.14	811.08	16.40
	HLA-DRB3*02:02	EYLDDRNTFRHSVVVPCE	18	NetMHCIIpan	32.56	659.15	16.40
	HLA-DRB1*04:05	LDDRNTFRHSVVVPC	15	Consensus	7.8	-	1.16
	HLA-DRB1*07:01	LDDRNTFRHSVVVPC	15	Consensus	5	-	12.21
	HLA-DRB3*02:02	LDDRNTFRHSVVVPC	15	NetMHCIIpan	9.1	563.59	16.40
	HLA-DRB1*07:01	LDDRNTFRHSVVVPCE	16	Consensus	6.46	-	12.21
	HLA-DRB3*02:02	LDDRNTFRHSVVVPCE	16	NetMHCIIpan	8.89	466.71	16.40
	HLA-DRB3*02:02	LDDRNTFRHSVVVPCEP	17	NetMHCIIpan	15.67	600.51	16.40
	HLA-DRB3*02:02	LDDRNTFRHSVVVPCEPP	18	NetMHCIIpan	32.56	695.28	16.40
	HLA-DRB1*04:05	NTFRHSVVVPCE	12	Consensus	9.79	-	1.16
	HLA-DRB1*07:01	NTFRHSVVVPCE	12	Consensus	9.19	-	12.21
	HLA-DRB3*02:02	NTFRHSVVVPCE	12	NetMHCIIpan	7.12	708.16	16.40
	HLA-DQA1*01:02/DQB1*06:02	NTFRHSVVVPCEP	13	Consensus	9.67	-	10.40
	HLA-DRB1*04:05	NTFRHSVVVPCEP	13	Consensus	7.64	-	1.16
	HLA-DRB1*07:01	NTFRHSVVVPCEP	13	Consensus	5.77	-	12.21
	HLA-DRB3*02:02	NTFRHSVVVPCEP	13	NetMHCIIpan	7.02	600.26	16.40
	HLA-DRB1*04:05	NTFRHSVVVPCEPP	14	Consensus	7.32	-	1.16
	HLA-DRB1*07:01	NTFRHSVVVPCEPP	14	Consensus	4.74	-	12.21
	HLA-DRB3*02:02	NTFRHSVVVPCEPP	14	NetMHCIIpan	7.43	556.15	16.40
	HLA-DRB1*04:05	NTFRHSVVVPCEPPE	15	Consensus	8.5	-	1.16
	HLA-DRB1*07:01	NTFRHSVVVPCEPPE	15	Consensus	5	-	12.21
	HLA-DRB3*02:02	NTFRHSVVVPCEPPE	15	NetMHCIIpan	9.3	575.94	16.40
	HLA-DRB1*07:01	NTFRHSVVVPCEPPEV	16	Consensus	6.46	-	12.21
	HLA-DRB3*02:02	NTFRHSVVVPCEPPEV	16	NetMHCIIpan	15.01	770.18	16.40
	HLA-DRB1*04:05	RNTFRHSVVVPC	12	Consensus	9.79	-	1.16
	HLA-DRB1*07:01	RNTFRHSVVVPC	12	Consensus	9.19	-	12.21
	HLA-DRB3*02:02	RNTFRHSVVVPC	12	NetMHCIIpan	6.23	629.95	16.40
	HLA-DRB1*04:05	RNTFRHSVVVPCE	13	Consensus	7.02	-	1.16
	HLA-DRB1*07:01	RNTFRHSVVVPCE	13	Consensus	5.77	-	12.21
	HLA-DRB3*02:02	RNTFRHSVVVPCE	13	NetMHCIIpan	3.9	350.48	16.40
	HLA-DQA1*01:02/DQB1*06:02	RNTFRHSVVVPCEP	14	Consensus	9.05	-	10.40
	HLA-DRB1*04:05	RNTFRHSVVVPCEP	14	Consensus	6.78	-	1.16
	HLA-DRB1*07:01	RNTFRHSVVVPCEP	14	Consensus	4.74	-	12.21
	HLA-DRB3*02:02	RNTFRHSVVVPCEP	14	NetMHCIIpan	3.98	294.56	16.40
	HLA-DRB1*04:05	RNTFRHSVVVPCEPP	15	Consensus	8.4	-	1.16
	HLA-DRB1*07:01	RNTFRHSVVVPCEPP	15	Consensus	5	-	12.21
	HLA-DRB3*02:02	RNTFRHSVVVPCEPP	15	NetMHCIIpan	5.2	310.03	16.40
	HLA-DRB1*07:01	RNTFRHSVVVPCEPPE	16	Consensus	6.46	-	12.21
	HLA-DRB3*02:02	RNTFRHSVVVPCEPPE	16	NetMHCIIpan	8.2	423.37	16.40
	HLA-DRB3*02:02	RNTFRHSVVVPCEPPEV	17	NetMHCIIpan	15.16	574.94	16.40
	HLA-DRB3*02:02	RNTFRHSVVVPCEPPEVG	18	NetMHCIIpan	32.56	674.71	16.40
	HLA-DRB1*07:01	TFRHSVVVPCEP	12	Consensus	9.19	-	12.21
	HLA-DRB1*07:01	TFRHSVVVPCEPP	13	Consensus	5.77	-	12.21
	HLA-DRB1*07:01	TFRHSVVVPCEPPE	14	Consensus	4.85	-	12.21

	HLA-DRB1*07:01	TFRHSVVVPCEPPEV	15	Consensus	5.1	-	12.21
	HLA-DRB1*07:01	TFRHSVVVPCEPPEVG	16	Consensus	6.46	-	12.21
	HLA-DRB3*02:02	VEYLDDRNTFRHSVVVPC	18	NetMHCIIpan	38.48	849.22	16.40
	HLA-DRB1*04:01	YLDDRNTFRHSVVVPC	16	Consensus	9	-	6.37
	HLA-DRB1*07:01	YLDDRNTFRHSVVVPC	16	Consensus	6.46	-	12.21
	HLA-DRB3*02:02	YLDDRNTFRHSVVVPC	16	NetMHCIIpan	12.7	679.25	16.40
	HLA-DRB3*02:02	YLDDRNTFRHSVVVPCE	17	NetMHCIIpan	15.84	607.86	16.40
	HLA-DRB3*02:02	YLDDRNTFRHSVVVPCEP	18	NetMHCIIpan	32.56	661.72	16.40
